# Spider: a flexible and unified framework for simulating spatial transcriptomics data

**DOI:** 10.1093/bioinformatics/btaf562

**Published:** 2025-11-14

**Authors:** Jiyuan Yang, Nana Wei, Yang Qu, Congcong Hu, Weiwei Zhang, Lin Liu, Hua-Jun Wu, Xiaoqi Zheng

**Affiliations:** School of Mathematical Sciences, Shanghai Jiao Tong University, Shanghai, 200240, China; Key laboratory of Carcinogenesis and Translational Research (Ministry of Education/Beijing), Department of Lymphoma, Peking University Cancer Hospital & Institute, Beijing, 100142, China; Center for Single-Cell Omics, School of Public Health, Shanghai Jiao Tong University School of Medicine, Shanghai, 200025, China; The Guangxi Key Laboratory of Intelligent Precision Medicine, Guangxi Zhuang Autonomous Region, Nanning, 530007, China; School of Mathematical and Information Sciences, Shaoxing University, Shaoxing, Zhejiang 312000, China; School of Mathematical and Information Sciences, Shaoxing University, Shaoxing, Zhejiang 312000, China; Institute of Natural Sciences, MOE-LSC, School of Mathematical Sciences, CMA-Shanghai, SJTU-Yale Joint Center for Biostatistics and Data Science, Shanghai Jiao Tong University, Shanghai, 200240, China; Key laboratory of Carcinogenesis and Translational Research (Ministry of Education/Beijing), Department of Lymphoma, Peking University Cancer Hospital & Institute, Beijing, 100142, China; Department of Biomedical Informatics, School of Basic Medical Sciences, Peking University Health Science Center, Beijing, 100191, China; Center for Precision Medicine Multi-Omics Research, Institute of Advanced Clinical Medicine, Peking University, Beijing, 100191, China; Center for Single-Cell Omics, School of Public Health, Shanghai Jiao Tong University School of Medicine, Shanghai, 200025, China; Hainan International Medical Center, Shanghai Jiao Tong University School of Medicine, Hainan, 571400, China

## Abstract

**Motivation:**

Spatial transcriptomics (ST) technologies provide valuable insights into cellular heterogeneity by simultaneously acquiring both gene expression profiles and cellular location information. However, the limited diversity and accuracy of “gold standard” datasets hindered the effectiveness and fairness of benchmarking rapidly growing ST analysis tools.

**Results:**

To address this issue, we proposed Spider, a flexible and comprehensive framework for simulating ST data without requiring real ST data as a reference. By characterizing the spatial patterns using cell type proportions and transition matrix between adjacent cells, Spider can produce more realistic and diverse simulated data and offer enhanced modeling flexibility compared to existing simulation methods. Additionally, Spider provides interactive features for customizing the spatial domain, such as zone segmentation and integration of histology imaging data. Benchmark analyses demonstrate that Spider outperforms other simulation tools in preserving the spatial characteristics of real ST data and facilitating the evaluation of downstream analysis methods. Spider is implemented in Python and available at https://github.com/YANG-ERA/Spider.

**Availability and implementation:**

All codes, simulated ST data in this paper are publicly available at https://github.com/YANG-ERA/Spider.

## 1 Introduction

Spatial transcriptomics (ST) has revolutionized our approaches to understanding cellular heterogeneity, the interplay between gene expression and cellular environment, and the location-specificity of gene expression within complex tissues (Rao *et al.* 2021, Moses and Pachter 2022, Palla *et al.* 2022, Williams *et al.* 2022). Unlike traditional single-cell RNA-sequencing (scRNA-seq) technologies which lose spatial information during cell dissociation (Kharchenko 2021), ST enables quantitatively measuring both the expression of individual RNA molecules and spatial information through spatially indexed barcode and next-generation sequencing techniques (Tian *et al.* 2023). Depending on the experimental assay employed, there are mainly two types of technologies for performing ST analysis (Rao *et al.* 2021). The first category is imaging-based technologies, which includes *in situ* hybridization [i.e. seqFISH (Eng *et al.* 2019), MERFISH (Chen *et al.* 2015), osmFISH (Codeluppi *et al.* 2018)], and *in situ* sequencing [i.e. STARmap (Wang *et al.* 2018) and FISSEQ (Lee *et al.* 2015)]. Although capable of profiling gene expression at single-cell or even subcellular resolution, these methods are restricted to preselected encoded probes and fail to achieve whole-transcriptome scale profiling (Zhang *et al.* 2022). The second category is based on next-generation sequencing (NGS) techniques, which encodes positional information onto transcripts before sequencing. Methods in this category include ST (Ståhl *et al.* 2016), 10x Genomics Visium (Rao *et al.* 2020), Slide-seq (Rodriques *et al.* 2019), Slide-seqV2 (Stickels *et al.* 2021), and Stereo-seq (Chen *et al.* 2022).

Based on ST data generated from the above techniques, researchers have proposed hundreds of downstream analysis tools (Zeng *et al.* 2022) to discover novel biological knowledge in plants (Lieben 2017), animals (Liao *et al.* 2021), and microbes (Dar *et al.* 2021) during the past few years. These tools were developed for a variety of tasks, including denoising (Liu *et al.* 2022, Wang *et al.* 2022), identifying spatially variable genes (Edsgärd *et al.* 2018, Svensson *et al.* 2018, Sun *et al.* 2020, Zhu *et al.* 2021, Zhang *et al.* 2022, Zhang *et al.* 2023), detecting spatial domains (Dong and Zhang 2022, Dries *et al.* 2021, Hu *et al.* 2021, Li and Zhou 2022, Teng *et al.* 2022, Yang *et al.* 2022, Zhao *et al.* 2021), inferring cell type compositions (for 10x Visium and Slide-seq) (Andersson *et al.* 2020, Elosua-Bayes *et al.* 2021, Song and Su 2021, Cable *et al.* 2022, Kleshchevnikov *et al.* 2022, Lopez *et al.* 2022, Miller *et al.* 2022, Sun *et al.* 2022, Hao *et al.* 2024), predicting the spatial distribution of RNA transcripts (Lopez *et al.* 2019, Welch *et al.* 2019, Abdelaal *et al.* 2020, Cang and Nie 2020, Biancalani *et al.* 2021, Shengquan *et al.* 2021, Hao *et al.* 2024), testing cell-cell/gene-gene interactions (Boisset *et al.* 2018, Arnol *et al.* 2019, Stuart *et al.* 2019, Tanevski *et al.* 2022, Pham *et al.* 2023), and imputing the missing spatial gene expression (Lopez *et al.* 2019, Abdelaal *et al.* 2020, Liu *et al.* 2020, Maseda *et al.* 2021, Shengquan *et al.* 2021, Hao *et al.* 2024).

However, the rapid advancement of ST sequencing technologies and analytical methods necessitates a re-evaluation and benchmarking of existing tools. This process requires either gold standard real datasets with known spatial domains or cell type annotation by pathologists, or well-conducted simulated datasets that resemble features of real data. Despite the accumulation of ST data in recent years, such as the mouse hypothalamus data (Moffitt *et al.* 2018) by MERFISH and human dorsolateral prefrontal cortex data (Maynard *et al.* 2021) by 10x Visium, researchers are still confronted with the following challenges: (i) Cell-type annotation by pathologists is not always accurate, as pathologists are often not able to effectively combine information from cellular phenotypes (e.g. cell size, shape, and nuclear density) and molecular profiles (Palla *et al.* 2022); (ii) The number and volumes of benchmark datasets are insufficient to validate the computational efficiency of different methods; (iii) Available benchmark datasets tend to prefer tissue samples with distinct layered structures, overlooking tumor samples showing spread or mutually exclusive patterns of infiltrated immune cells (Yuan *et al.* 2023). These tumor samples are difficult to manually annotate but may hold greater value in clinical contexts. Therefore, utilizing simulated data may be a feasible alternative option to overcome the above challenges and boost the development and evaluation of ST methods (Zhu *et al.* 2023, Song *et al.* 2024).

Motivated by simulators developed for other types of biological data (such as bulk and single-cell RNA-seq data), we distilled four critical criteria for an ideal ST data simulator: (i) Biological authenticity: Simulated data should follow authentic biological patterns and rules that govern cellular spatial organization, gene expression gradients, and tissue architecture; (ii) Flexibility: The simulator should be able to generate simulated data with diverse spatial patterns, not necessity cell type arrangement from the reference dataset. (iii) Reproducibility: The simulator should be robust to input variations and capable of consistently reproducing results. (iv) Practicality: The simulator should be user-friendly for individuals lacking domain knowledge. To the best of our knowledge, none of the existing simulators meet all four criteria simultaneously.

To address the above criteria, we here developed Spatial Transcriptomic Data Simulator (Spider), a Python-based software for flexibly, reproducibly, and practically simulating ST datasets. A key feature of Spider is its use of cell type proportions and transition matrix of adjacent cells to characterize the spatial pattern of simulated data, without requiring real ST data as reference. This approach enables Spider to generate more diverse spatial patterns in the simulated data, such as immune hot/cold tumor samples and stratified tissue samples with diverse cell type compositions. Furthermore, Spider provides an implementation interface for most existing simulation methods and a collection of available gold-standard datasets to facilitate researchers in promptly characterizing, benchmarking, and evaluating new analytical methods.

## 2 Methods

### 2.1 Cell type assignment

Spider is designed to simulate ST data with a given spatial pattern. It first generates single-cell resolution ST data, and then aggregates to spot-level under a given gridding scheme. The single-cell resolution ST data is generated as follows: given the number of cells N, Spider randomly generates spatial coordinates of individual cells under a 2D/3D Uniform distribution, which denote as $S={\left(s_{1},\ldots ,\;s_{N}\right)}^{T},\;s_{i}\in R^{2}/R^{3}$. Then, it assigns cell type labels to each individual cell locations to approximate the given spatial pattern, and randomly extracts simulated (or real) single-cell data from the corresponding cell type as expression profile for each cell location. Among the above steps, the cell type assignment step is the most critical and computational challenging. We formulated it as a constrained binary optimization problem as follows.

Spider takes pre-set proportion of cell types $\pi ={\left(\pi_{1},\ldots ,\pi_{K}\right)}^{T}\;$and transition probabilities between cell types $P\in R^{K\times K}$ as input, where K is the number of cell types. The output cell type label matrices $X\in R^{N\times K}$ should satisfy that the transition probability between adjacent cell types and their proportion after cell type assignment should be as close as possible to the given one. Thus, we have the following optimization objective:


$$\arg\underset{X}{\min}{\left\|P-C^{-1}X^{T}AX\right\|}_{F},$$

s.t.{Xij∈{0,1}, i=1,…,N,j=1,…,K,X1K=1N,XT1N=π×N,

where A is the adjacency matrix of the graph built by spatial coordinates S of cells (see Spatial neighborhood graph construction step below). $X^{T}AX$ is the transition frequency matrix based on the cell type assignment $X,\;C=\mathrm{diag}(X^{T}AX1_{K})\;$is the row-normalized factor matrix, and $X_{ij}$ is an indicator variable of the cell i to cell type j. $1_{K}$ denotes a vector of K ones, with $1_{N}$ similarly defined.

### 2.2 Batched simulated annealing

To solve the aforementioned constrained binary optimization problem efficiently for large-scale simulation, we developed a batched simulated annealing algorithm (BSA) modified from the simulated annealing algorithm. BSA aims to find an acceptable solution for the problem by leveraging the benefits of the conventional simulated annealing while alleviating its computational burden via a parallel computing technique (Fig. 2B).

BSA starts with a cell plate, which could be a rectangle in a two-dimensional space. Given an input cell number, the spatial coordinate of each cell is generated from a 2D uniform distribution across the cell plate by default. Then, the cell plate is partitioned into multiple identical grids, termed cell zones, which contain varying numbers of cells. The number of initial cell zones is set to the square root of the total cell number by default to evenly partition the rows and columns of cell plate. Next, BSA splits cell plate into cell zones with different window sizes. Through gradually decreasing the window sizes from large to small, BSA produces a series of refined cell zones. BSA performs simulated annealing on the cell zones extracted at each epoch rather than individual cells to improve efficiency. In detail:

1. Initialization and partition. BSA recursively partitions the cell plate into cell zones with shrinking window sizes. We set the total number of epochs $M=\left\lfloor {\frac{1}{2}\log}_{2}\frac{N}{1000}+1\right\rfloor$, where $\left\lfloor \cdot \right\rfloor$ denotes the floor function. In the m-th epoch, the length and width of the extracted windows are set as $l_{m}=l_{0}*2^{M-m}$ and $w_{m}=w_{0}*2^{M-m}$ respectively, where $l_{0}$ and $w_{0}$ are the length and width of the initial cell zones. After each partition epoch, BSA assigns the cell type label of each cell zone by a multinomial distribution based on the initial cell type proportions *π* at the first epoch. To accelerate the convergence, the number of cell type swaps per simulated annealing iteration is also increased exponentially across epochs as ${\mathrm{swap}}_{m}=2^{m}$.
2. Simulated annealing on cell zones. Spider performs simulated annealing on cell zones in each partition epoch. For each new cell zone generated in an epoch, we assigned it with the cell type label that inherits from its parent zone in previous epoch. Through this hierarchical label inheritance step, the transition matrix of cell types in the initial cell zone converges to the pre-set transition matrix as the number of epochs increase.
3. Cell type assignment and adjustment in single-cell level. BSA assigns label of each cell zone to its housed cells. Since the cell type proportions in each epoch is not faithfully held after cell zone partition, we recalibrated the cell type assignment by randomly switching a small proportion of surplus and deficient cell types until reaching the pre-set proportion.

### 2.3 Predefined spatial pattern configuration

We set some predefined spatial patterns in Spider, including attractive, repulsive, layered and gyrus. These patterns represent distinct cellular organizations commonly observed in complex tissues:

The attractive mode indicates that each cell type exhibits a clustered spatial tendency. We implemented this by setting the diagonal elements of the cell type transition matrix to a high probability (0.8), with uniform transition probabilities to all other cell types.

The repulsive mode indicates that each cell type demonstrates a preference for remaining within its own group while avoiding other cell types. Within each group, cell types have a high probability of transitioning to themselves or their group partner, while transitions between groups are minimized. This arrangement simulates biological scenarios where functionally related cell types cluster together, creating distinct spatial compartments within the tissue.

The layered mode organizes cell types into distinct layers, encouraging intra-layer interactions while minimizing inter-layer interactions. In this mode, we generated spatial patterns of cell types according to a multinomial distribution, where each layer has a dominant cell type with the probability distribution parameter of 0.8.

The gyrus mode indicates that cell types are organized in a convoluted spatial arrangement, analogous to the folds of a gyrus in the brain. This mode enhances interactions between neighboring cell types, fostering complex interplay and localized clustering while maintaining distinct boundaries. While its generation method is similar to the layered mode, the gyrus mode emphasizes a more intricate, convoluted structure that promotes interactions among neighboring cell types.

### 2.4 Spatial neighborhood graph construction

Spatial neighborhood graph reflects the complex spatial interactions between cells. According to different sequencing platforms adopted, Spider provides various metrics to construct spatial neighborhood graph of cells based on spatial coordinate information S. For NGS-based platform which generates the grid-like spot arrangement types, such as hexagonal grid for 10x Visium and square grid for ST platform, Spider constructs k-nearest neighbor (kNN) graph based on Euclidean distance of these data, where k is the number of neighbors. Then, a sparse adjacent matrix A based on kNN graph can be calculated as:


Aij={1, j∈N(i),0,otherwise,

where N(i) represents the set of neighbors of cell i.

For image-based techniques that has generic spot coordinates of cells, Spider provides a user-specific parameter r to measure the radius of each cell. Then adjacent matrix A is calculated as:


Aij={1, d(i,j)<r,0,otherwise,

where $d(i,j)={\left\|s_{i}-s_{j}\right\|}_{2}$ is defined as the Euclidean distance between cell i and cell j. Besides Euclidean distance, Spider also implements other distance metrics, such as cosine and Manhattan distances, in construction of the kNN and spatial neighborhood graphs.

### 2.5 Benchmark metrics

The following measurements are used to evaluate the performance of the simulation methods.

#### 2.5.1 Clustering accuracy

Adjusted Rand index (ARI) (Hubert and Arabie 1985) and normalized mutual information (NMI) (Emmons *et al.* 2016) are used to measure the clustering accuracy of different methods. Given two sets of clustering labels, the ARI is calculated as:


ARI= ∑i,j(nij2)-(∑i(ni2)∑j(nj2))/(n2) 12(∑i(ni2)+∑j(nj2))-(∑i(ni2)∑j(nj2))/(n2),

where $n_{ij}$ is the number of spots overlapped by cluster i and cluster j. $n_{i}$ and $n_{j}$ are the number of spots in cluster i and j, respectively. NMI is calculated as:


$$\mathrm{NMI}=\;\frac{\sum_{i,j}p_{ij}\log\frac{p_{ij}}{p_{i}p_{j}}\;}{(-\sum_{i}p_{i}\log p_{i}-\sum_{j}p_{j}\log p_{j})/2},$$

where $p_{ij}=\frac{n_{ij}}{n}$, $p_{i}=\frac{n_{i}}{n}$ and $p_{j}=\frac{n_{j}}{n}$.

#### 2.5.2 Spatial pattern metrics

We used three matrices, i.e. transition matrix (TM), centralized score matrix (CSM), and neighborhood enrichment matrix (NEM), to characterize the global spatial pattern of an ST data (Teshale *et al.* 2016, Behanova *et al.* 2022). CSM is used to describe complex relationships in large networks and can be represent as:


$$\mathrm{CSM}=[G_{1},G_{2},G_{3}]\in R^{K\times 3},$$

where $G_{1}$ is group degree centrality, which measures the ratio of non-group members that are connected to group members, $G_{2}$ is the average clustering coefficient measuring how likely the cluster nodes favor to cluster together, and $G_{3}$ is the group closeness centrality is defined as the normalized inverse sum of distances from the group to all nodes outside the group.

NEM quantifies the enrichment between each pair of cell types (Schapiro *et al.* 2017). Let $x_{ij}$ be the number of connections between cluster i and cluster j. Through preserving the spatial structure and shuffling the cell type labels m times, and then recounting the number of connections between each pair of clusters, a z-score can be calculated as:


$${\mathrm{NEM}}_{ij}=\frac{x_{ij}-\mu_{ij}}{\sigma_{ij}},\;i,j=1,\ldots ,\;K,$$

where $\mu_{ij}$ is the expected means and $\sigma_{ij}$ is the standard deviations for each pair in the random configuration.

We calculated the above three matrices for both simulated and reference data respectively, and the Frobenius norms between two matrices are treated as the spatial pattern discrepancy between simulated and real data.

#### 2.5.3 Cell-cell communication metrics

We calculated the similarity index (SI) and rank-based similarity index (RSI) (Luo *et al.* 2023) to examine the overlapping fraction between two sets of predicted ligand-receptor (LR) pairs. SI is a modified version of Jaccard index, which is designed to detect overlapping ratio of LR pairs between two cell-cell communication tools after correcting the quantity difference:


$$SI=\frac{\left|A\cap B\right|}{\min(|A|,|B|)},$$

where A (and B) represents the set of LR pairs inferred by method A (and B), and A∩B represents their overlapping set. |·| denotes the number of elements in a set.

RSI is defined to assess whether the overlapping LR pairs between different methods exhibit similar rankings among all LR pairs predicted by each method:


$$\mathrm{RSI}=1-\frac{1}{\left|C\right|}{\left\|\frac{R_{A}^{C}}{\left|A\right|}-\frac{R_{B}^{C}}{\left|B\right|}\right\|}_{L^{1}}=1-\frac{1}{\left|C\right|}\sum_{i=1}^{\left|C\right|}{\left\|\frac{R_{A}^{i}}{\left|A\right|}-\frac{R_{B}^{i}}{\left|B\right|}\right\|}_{L^{1}},$$

where C=A∩B represents the overlapping set of LR pairs, $R_{A}^{C}$ and $R_{B}^{C}$ represent the ranks of overlapping LR pairs based on the predicted LR scores in A and B, respectively. $R_{A}^{i}$ or $R_{B}^{i}$ represents the *i*-th element of $R_{A}^{C}$ or $R_{B}^{C}$. ${\left\|.\right\|}_{L^{1}}$ represents the $L^{1}$ norm.

### 2.6 Implementations of different simulation methods

All ST simulators are implemented according to the instructions in their original publications to generate simulation data using STARmap data as reference. To be specific,

RCTD and stereoscope. For each spot, cell types are randomly selected from the reference data and their proportions are sampled from the Dirichlet distribution. The gene expression profile of each spot is calculated as the sum of all cells in that spot. In order to get cell-level data, the number of cells per spot is set to 1.

STRIDE. Each spot is a mixture of cells randomly selected from STARmap data. It uses predefined minimum and maximum numbers of cell types in each spot. Similar to RCTD and stereoscope, the cell-level data are generated by setting the number of cells per spot to 1.

FICT. The cell type in each cell location is assigned via Gibbs sampling based on a prior transition matrix. Gene expression is then randomly sampled from the reference data.

STdeconvolve. Based on single-cell resolution ST data, spot-level data is obtained by dividing the space into grids and then merging cells within each grid, thereby inheriting the spatial information from the original ST data.

SpatialPCA. It begins with a reference tissue with known layered structure (DLPFC is adopted in our simulation). A number of pixels are then randomly sampled from the H&E image to serve as cell locations. Cell type in each cell location is then assigned according to the cell types proportion in the corresponding layer, and gene expression for each cell is simulated from the Splatter package under default parameters.

SRTsim. SRTsim simulates expression counts for individual genes by utilizing count models learned from the reference data. It assigns the simulated counts to spatial locations in the simulated data while retaining the original spatial expression pattern observed for each gene in the reference. SRTsim provides two options for its reference-based simulation module: SRTsim_tissue and SRTsim_domain. In SRTsim_tissue, expression counts are directly simulated for all spatial locations across the entire tissue. In SRTsim_domain, expression data are first simulated separately within each tissue domain and then combined across domains. In addition, SRTsim also enables reference-free simulation where spatial patterns can be designed either from a customized shape or user-specified model parameters.

scDesign3. scDesign3 incorporates cell state covariates including cell type, pseudo time, batch effects, and condition effects as a generalized additive model. For simulation, scDesign3 first estimates gene expression distributions based on the reference data and generates new expressions from the distributions.

scCube. scCube consists of two main components: gene expression simulation and spatial pattern simulation. For gene expression simulation, it employs a variational autoencoder (VAE) model to generate gene expression profiles for different cell or spot populations. The spatial pattern simulation offers two strategies: a reference-based approach that uses optimal transport to map generated cells/spots to spatial locations, and a reference-free approach that can generate random spatial patterns based on multivariate normal distribution to simulate spatial correlation or customized spatial patterns, such as cell clusters, ring structures, vessel-like structures, etc

## 3 Results

### 3.1 Summary of existing ST simulation tools

**Figure 1.**
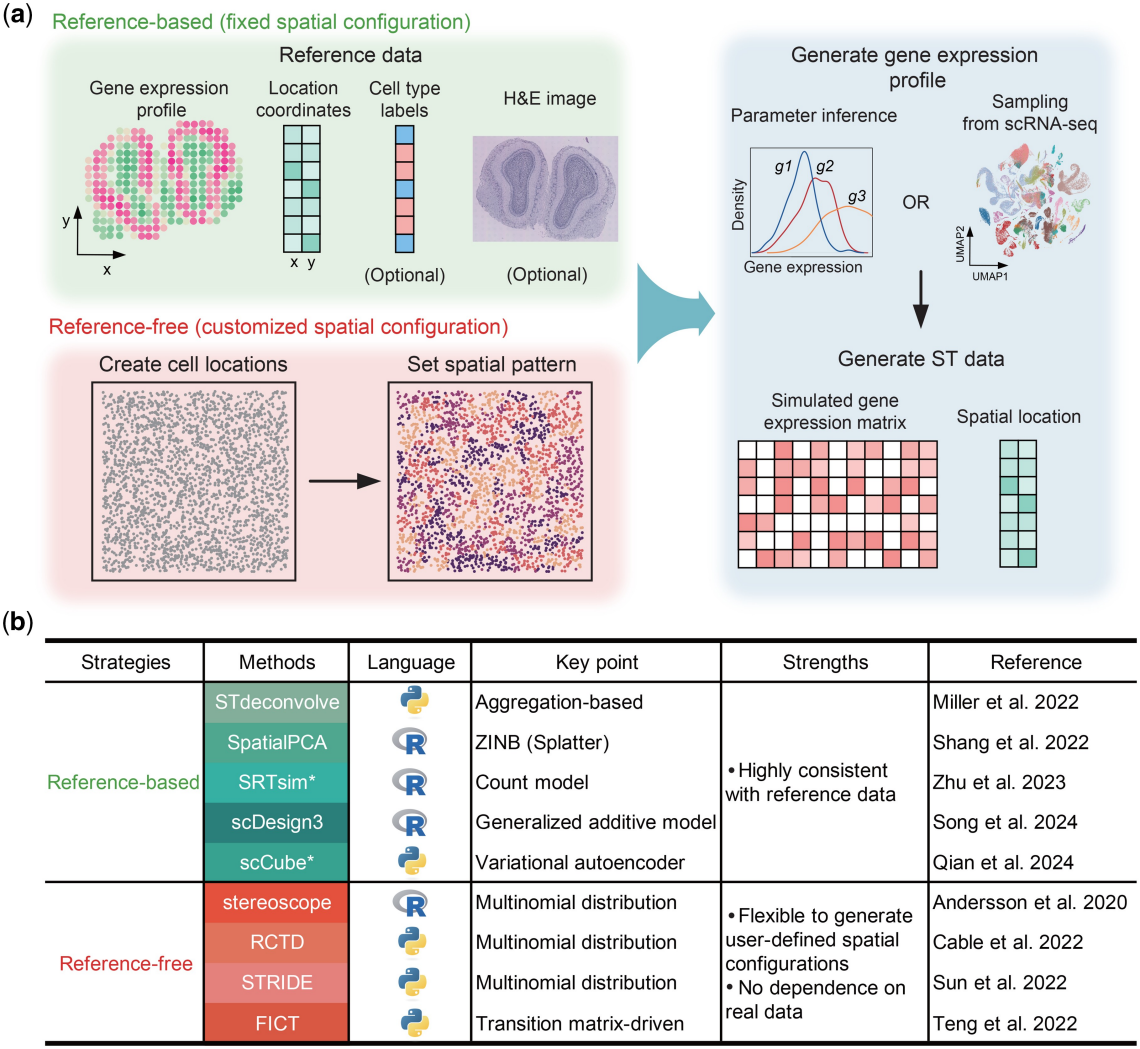
Summary of existing simulation methods. (A) Schematic overview of the ST data simulation workflow (reference-free and reference-based). The key distinction between the reference-free and reference-based simulation strategies lies in their respective input data requirements (red panel). In the reference-free approach, the simulation workflow begins with generating a cell plate without pre-assigned cell type information. This blank cell plate is then populated with diverse cell type assignments to establish the desired spatial patterns. The gene expression profiles are subsequently simulated or sampled from matched scRNA-seq data based on the generated cell types and spatial topology (blue panel). In contrast, the reference-based strategy relies on the availability of an existing spatial transcriptomics dataset as a reference (green panel). This reference data is used to estimate the statistical parameters governing the gene expression distributions within the tissue (blue panel). Some methods may also require matched H&E image as additional information to guide the simulation process. The simulators marked with an asterisk (*) have both reference-free and reference-based versions. (B) Summary of nine simulation methods grouped by two simulation categories, focusing on the key characteristics of each simulation method, including the programming language, key point, strengths, and reference.

Existing ST simulation tools can be broadly divided into two major categories based on their underlying principles: reference-free and reference-based methods (Fig. 1A). These complementary simulation strategies allow researchers to select the most appropriate approach based on the availability of reference data and the specific objectives of their spatial transcriptomics studies.

Reference-based methods directly inherit spatial topology of cell types from reference data. These methods primarily require real spatial transcriptomics data as mandatory input to extract the spatial coordinates of spot/cell locations, along with the corresponding gene expression matrix. When cell type annotations are available, statistical models (e.g. hierarchical Bayesian frameworks, conditional auto-regressive models) are employed to infer cell type-specific expression parameters (mean, dispersion, inter-gene covariance) for each cell type. Some methods in this category may also require a matched H&E image as additional information to further guide the simulation (Shang and Zhou 2022). This dependence on spatial templates ensures simulations with high contextual fidelity, but constrains them to the biological variability captured within specific reference inputs (Fig. 1B). Typical examples in this category include STdeconvolve (Miller *et al.* 2022), SpatialPCA (Shang and Zhou 2022), SRTsim (Zhu *et al.* 2023), scDesign3 (Song *et al.* 2024), and scCube (Qian *et al.* 2024).

In contrast, reference-free simulation approaches do not rely on existing spatial transcriptomics data. Instead, these algorithms generate spatial frameworks *de novo* by establishing synthetic cellular coordinates, followed by algorithmic assignment of cell type identities according to user-specified spatial distribution rules. After spatial configuration, Gene expression profiles are then generated through either: 1) parametric sampling from defined statistical distributions (e.g. negative binomial, zero-inflated models), or 2) template-based sampling from available scRNA-seq datasets. The key advantage of this approach lies in its high flexibility, which allows users to create diverse spatial patterns and benchmark scenarios according to their research objective (Fig. 1B). Examples in this category include stereoscope (Andersson *et al.* 2020), RCTD (Cable *et al.* 2022), STRIDE (Sun *et al.* 2022), and FICT (Teng *et al.* 2022) (Fig. 1B). Note that these tools are not designed specifically for simulation purpose but for benchmarking their downstream methods such as cell type clustering (e.g. FICT) and deconvolution (e.g. stereoscope, RCTD and STRIDE). We therefore included them as valid comparative benchmarks to demonstrate the methodological spectrum of reference-free implementations. Although achieving substantial improvements, these tools still suffer from significant limitations, particularly in inadequately leveraging spatial patterns and dependencies to empower simulated data, which restrict their ability to capture the complex, multi-scale spatial organization of real tissues. It is worth mentioning that although two reference-based simulators, SRTsim and scCube, also offer a reference-free mode, their implementation is either through a simply interactive interface or requires manual annotation of the spatial domain, limiting their ability to efficiently produce diverse spatial patterns compared to the typical reference-free methods.

### 3.2 Overview of Spider

**Figure 2.**
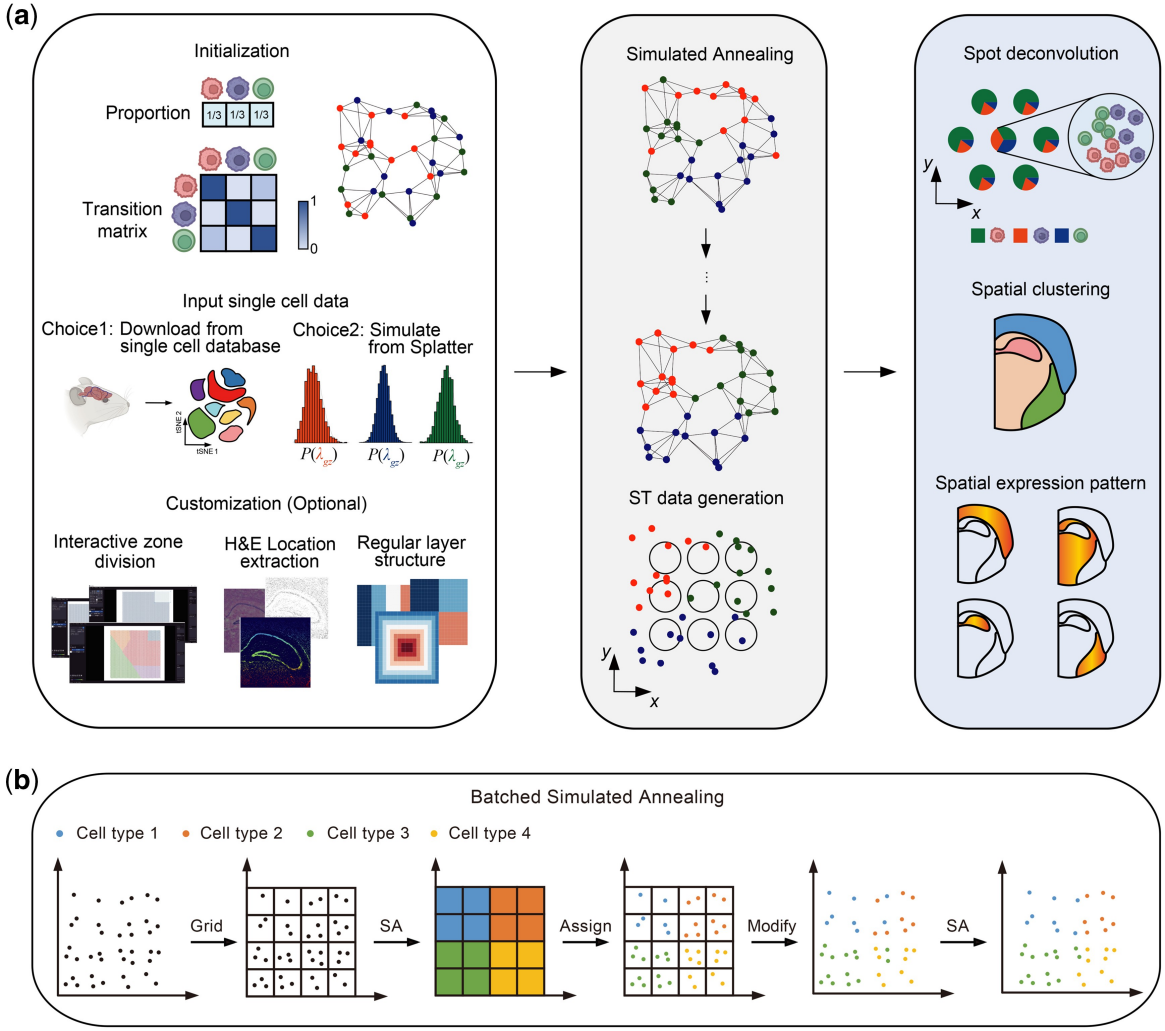
Framework of Spider. (A) Spider takes input parameters including the number of cell locations in ST data plate, the number of cell types, their prior proportions, and a transition probability adjacency matrix. These parameters inform the construction of a spatial neighbor network (the top-left panel). Single cell expression profiles can be either obtained from real data or simulated using tools such as Splatter (the mid-left panel). Next, employing a batched simulated annealing algorithm, Spider iteratively adjusts cell type labels within the spatial neighbor network to approximate the targeted transition matrix (top of middle panel). Then, gene expression profiles of all cells in each spot are summed up to obtain the spot-level ST data (the bottom-mid panel). Given simulated ST data by Spider, one can evaluate downstream analytical methods including spatial deconvolution, clustering and identification of spatially variable genes (right panel). In addition, Spider also supports interactive zone division and cell location information extraction from H&E image, and offers a variety of standard layer structures (bottom of left panel). (B**)** Batched simulated annealing algorithm used in Spider. Cell plate is first split into regular grids, and cell type labels are randomly assigned to each grid based on the prior cell type proportions; Spider treats each grid as a representative unit of the cells in that area. Spider views all representative units as a batch and performs the simulated annealing algorithm at the grid-level; After convergence, Spider retraces and assigns the label of the grid to all cells in that grid; Finally, Spider performs the simulated annealing algorithm at the cell-level to refine the optimization.

To address the above shortcomings, we developed Spider, a universal reference-free simulation framework for ST data capable of generating diverse cellular spatial patterns based on either user-defined spatial dependence parameters or predefined spatial patterns (attractive, repulsive, layered and gyrus, see Methods for details). The implementation of Spider consists of three main steps: (1) Initializing model parameters, (2) Assigning cell types and allocating gene expression, and (3) Aggregating gene expression to the spot level (optional for spot-level ST data) (Fig. 2A).

In the first step, Spider takes user-specified parameters as input, including the number of cell locations on a simulated plate, the number and proportion of cell types, and a transition matrix between cell types, along with matched gene expression profiles. Note that some of these parameters, such as the transition matrix, can be simplified by a predefined spatial pattern (e.g. attractive, repulsive, layered and gyrus). Gene expression profiles can be obtained from either a real scRNA-seq data or scRNA-seq simulation tools such as Splatter (Zappia *et al.* 2017) or scDesign2 (Sun *et al.* 2021). Then, Spider generates cell locations in the plate either randomly or in a uniform grid-like pattern, and constructs a neighborhood graph of cell locations according to their pairwise distances on the plate. Spider supports various strategies in constructing cell neighborhood graph, such as k-nearest neighbors (kNN) (Zhang 2016), shared nearest neighbors (SNN) (Ertoz *et al.* 2002), or neighbors identified by Delaunay triangulation (Lee and Schachter 1980, Palla *et al.* 2022).

In the second step, Spider assigns cell type labels to each cell location to approximate the user-specified spatial pattern, described by cell type proportions and the pairwise transition probabilities between cell types on the cell neighborhood graph. We formulated this step as a binary optimization problem with integer constraints (see Methods for details). However, since this is a combinatorial optimization problem, it is challenging to solve in polynomial time, especially for large scale simulations. Therefore, we proposed three heuristic strategies based on the simulated annealing (SA) algorithm (Kirkpatrick *et al.* 1983): global simulated annealing (GSA), local simulated annealing (LSA), and batched simulated annealing (BSA) (Fig. 2B, Supplemental Fig. S1). GSA algorithm directly performs SA at the cell level, which is suitable for generating small samples with less than 1000 cells. LSA splits the cell plate into regular grids, and performs SA for each grid in parallel (Supplemental Fig. S1), which preserves a high degree of spatial patterns and structures for each cell type. BSA partitions the cell plate into several patches and employs the SA algorithm to optimize the transition matrix for each patch. As the patch size decreases, the global transition matrix gradually converges to the target, leading to a significant enhancement in convergence speed (Supplemental Fig. S2).

In the final step, Spider generates the simulated ST data at the spot level by aggregating gene expressions of all cells within each spot. Users can specify the shape (circle or rectangle) and size of spots to generate a regular array of spatial spots, then the expression profiles of all captured cells within each spot are summed up to obtain the spot-level expression profiles.

In addition to the core simulation functionality, Spider also supports optional interactive operations, including domain segmentation, extraction of cell coordinates from histology (H&E) images, and customization of domain structures (Supplemental Fig. S3). For interactive domain segmentation, Spider can manually delineate the spatial domain of simulated cells using *Napari*, a multi-dimensional image viewer in Python. Spider can also take H&E staining images as input to generate cell coordinates using an anatomical segmentation algorithm Cellpose (Stringer *et al.* 2021), which is specifically designed for nuclei segmentation. Furthermore, Spider provides several customized regular spatial domain structures for users, such as striped, blocked, and gyrus (Supplemental Fig. S3C–E). To enable researchers to utilize different simulation methods within a unified framework, we have also integrated most of the above reference-free simulators into Spider. For example, one can simply implement RCTD, STRIDE, and FICT using the functions *spider.rctd, spider.stride, and spider.fict*, respectively.

To elucidate the impact of parameter settings on the generated spatial patterns, we performed exploratory analyses by simulations using two cell types as illustrative examples. Here, we fixed the proportions of two cell types at 0.8 and 0.2, and varied their corresponding transition probabilities across a range from 0 to 1 with a step size of 0.1. This simulation revealed three typical patterns: mixed, compartmentalized, and layered structures, respectively (Supplemental Fig. S4). When the transition probabilities within the same cell type are low (e.g. below 0.3) yet the transition probabilities between two cell types are relatively high, cells tend to intermingle in patches, forming mixed patterns (Supplemental Fig. S4A). As the transition probability within a cell type increases, cells of this cell type gradually aggregate. Concurrently, high transition probability between two cell types results in circular surrounding structures (compartmentalized, Supplemental Fig. S4B). Finally, in case of high within-cell-type and low between-cell-types transition probabilities, cells from the same cell type both aggregate and one cell cluster gathers at the periphery, generating a layered structure (Supplemental Fig. S4C). A similar trend is observed when extending the analysis to three cell types (Supplemental Fig. S5). Overall, by adjusting transition probabilities of within or between cell types, users can generate desired cell distribution patterns in the simulated data.

### 3.3 Performance comparison of ST simulation tools

**Figure 3.**
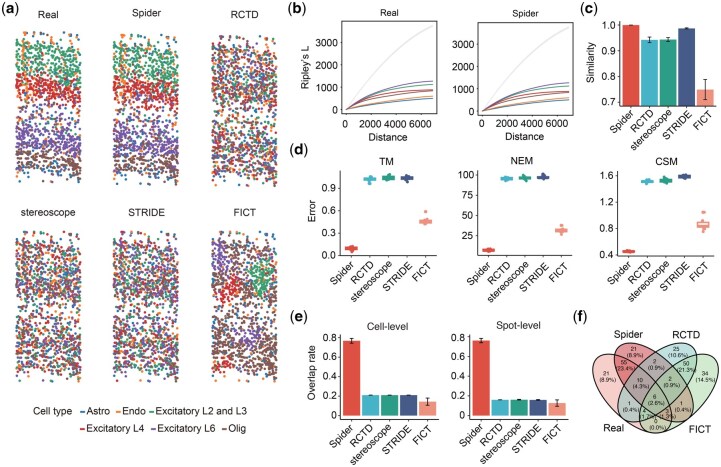
Comparison of ST simulation tools based on the STARmap reference data. (A) Cell type distribution of the real STARmap dataset and four simulated results by Spider, RCTD, stereoscope, STRIDE, and FICT. (B) Ripley’s *L* curves illustrating the global spatial patterns of each cell type in the reference and simulated data by Spider. The *x*-axis represents the spatial distance at which the Ripley’s *L* function evaluates the spatial pattern of each cell type. The shaded gray line represents the expected *L*-function of a random pattern under spatial Poisson point process. (C) Cosine similarity of the Ripley’s *L* curve between the reference and simulated data generated by different simulation methods. (D) Differences between real and simulated data in terms of three spatial pattern matrices (i.e. TM: transition matrix, CSM: centrality scores matrix, NEM: neighborhood enrichment matrix). (E) Accuracy of SVG detection on simulated data generated by different simulation methods. Error bars indicate 95% confidence intervals across 10 replicates. (F) Venn diagram showing the overlap of SVGs identified by SPARK in real data and simulated data generated by different simulation methods at cell level.

Compared with other reference-free simulation tools, Spider also facilitates parameter estimation (including cell type proportions and transition probabilities) directly from real spatial transcriptomics data. This functionality allows Spider to incorporate information derived from reference datasets, thereby enhancing its ability to accurately capture the intrinsic spatial characteristics of various cell types. To demonstrate the effectiveness of Spider, we conducted a comparative analysis against four reference-free simulators: RCTD, stereoscope, STRIDE, and FICT. Our evaluation focused on their respective agreements with real ST data, in terms of the overall cell type distribution pattern and several spatial consistency metrics. Specifically, we utilized single-cell resolution mouse visual cortex data that captured 1,549 cells, representing 15 cell types identified through clustering with carefully curated marker genes (Supplemental Fig. S6) (Wang *et al.* 2018). To ensure fair comparisons, we excluded ambiguous cell types and retained the six most abundant cell types with sufficient cell numbers as cell-level reference data. The final dataset consists of 1234 cells including 141 astrocytes, 150 endothelial cells, 258 excitatory L2/3 cells, 198 excitatory L4 cells, 287 excitatory L6 cells, and 200 oligodendrocytes (Fig. 3A). Then, we gridded this single-cell resolution data into 500×500 squared pixels, yielding 364 spots, each covering 1–10 cells. Taking this dataset as a reference, we used location information of cells and gene expression profiles sampled from the real dataset as inputs to each method. For Spider and FICT, we also estimated cell type proportions and transition matrix as additional inputs (Supplemental Fig. S7). Each simulation method is repeated 10 times to assess the robustness.

We evaluated the performance of different methods in terms of consistency with real ST data, focusing on cellular spatial patterns and gene expression spatial patterns. The real dataset shows excitatory neuron cells clustered separately in Layers 2/3, 4, and 6 (Fig. 3A). This spatial clustering pattern is exclusively observed in the simulated dataset by Spider (Fig. 3A, Supplemental Fig. S8). Moreover, Spider also preserves spatial gene expression patterns as demonstrated by marker genes (Supplemental Fig. S9). Other methods lead to severely biased cell type proportions compared to the real dataset (Supplemental Figs. S10–S13, Table S1).

To quantify the similarity of the distributions of all cell types, we plotted the Ripley’s *L* curve for each method, which enables the detection of the spatial patterns (i.e. random, clustered, and dispersed) of the six cell types (Fig. 3B) (Haase 1995). All cell type clusters simulated by Spider are below the baseline (grey line), indicating a dispersed pattern across the area, which is consistent with the reference dataset. Although the cell type clusters simulated by RCTD, stereoscope, and STRIDE are also below the baseline, most of their cell types show significantly different trends in Ripley’s *L* value compared to the reference dataset as the distance increases (Supplemental Figs. S14 and S15). We further computed the cosine similarity of Ripley’s *L* curve between the real and simulated datasets generated by different simulation methods. Spider achieves the best performance (mean: 0.999) compared to other methods (Fig. 3C).

In addition, we demonstrated Spider’s robustness to spatial coordinate perturbation. We randomly generated new cell coordinates within each tissue domain from uniform distributions, while maintaining the original cellular compositions. Using the same evaluation criteria, Spider produces results comparable to those from the fixed coordinate scenario, indicating that it captures underlying biological organizational principles rather than simply replicating static spatial configurations (Supplemental Fig. S16).

We subsequently evaluated the spatial consistency between simulated data produced by each method and the reference data in terms of the transition matrix (TM), centrality scores matrix (CSM) and neighborhood enrichment matrix (NEM) (Fig. 3D). The errors of each method are calculated as the distance between real and estimated matrices across 10 replicates. Among all tested methods, Spider consistently exhibits the lowest error between simulated and real data (Fig. 3D).

Finally, we compared different methods in recovering spatially variable genes (SVGs). We selected the top 100 genes with the lowest adjusted *P*-value identified by SPARK (Sun *et al.* 2020) from the real data as reference. We then computed the power value, which measures the overlap ratio between SVGs identified from each simulated data and those identified from the real data. In both cell-level and spot-level, Spider achieves superior performance compared to other methods (Fig. 3E). Venn diagram of the detected SVGs by different methods on cell level also demonstrates the highest overlap of Spider with the reference data (Fig. 3F). Overall, Spider achieves better performance than other competitor methods, indicating its ability to effectively and robustly preserve the spatial pattern of the reference data.

### 3.4 Benchmarking downstream analysis using simulated ST data

**Figure 4.**
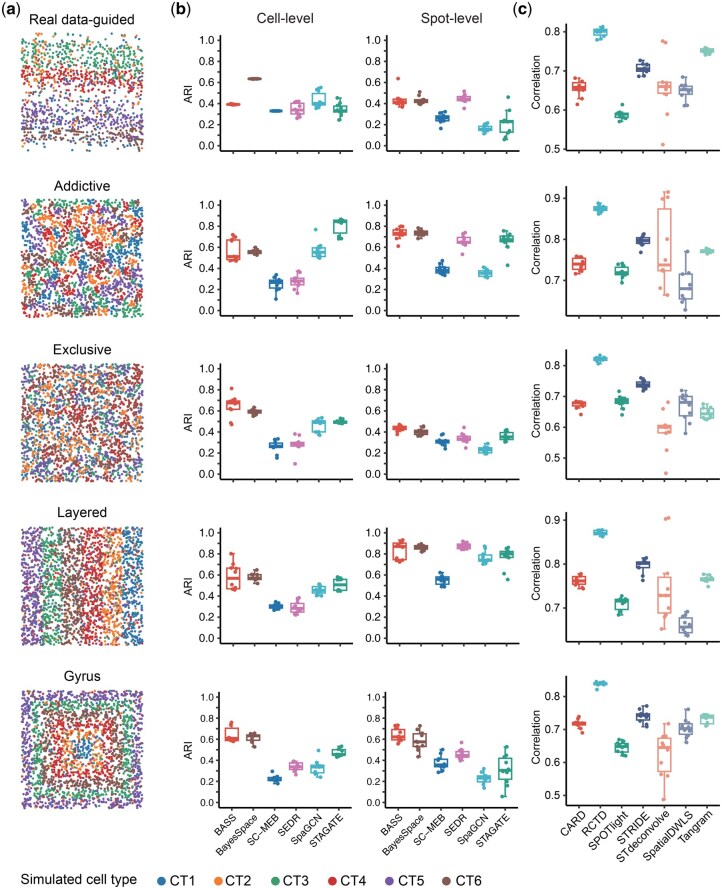
Evaluation of different simulation tools using downstream analyses. (A) Distribution of five different spatial patterns simulated by Spider. (B) Accuracy of six clustering methods (*x*-axis) across the five simulated datasets. (C) Correlation between the estimated cell-type compositions by seven deconvolution methods and ground truth.

To demonstrate the utility of Spider in downstream analyses, we simulated five datasets with different spatial patterns, including real data-guided, attractive, repulsive, layered, and gyrus (Fig. 4A), to evaluate the performance of different ST analysis tools. We focus mainly on two types of downstream analysis tasks: spatial domain detection (spatial clustering) and spot deconvolution. Six clustering algorithms (BASS (Li and Zhou 2022), BayesSpace (Zhao *et al.* 2021), SC-MEB (Yang *et al.* 2022), SEDR (Xu *et al.* 2024), SpaGCN (Hu *et al.* 2021), and STAGATE (Dong and Zhang 2022)) and seven deconvolution methods (CARD (Ma and Zhou 2022), RCTD (Cable *et al.* 2022), SPOTlight (Elosua-Bayes *et al.* 2021), STRIDE (Sun *et al.* 2022), STdeconvolve (Miller *et al.* 2022), SpatialDWLS (Dong and Yuan 2021), and Tangram (Biancalani *et al.* 2021)) are selected for benchmarking task due to their popularity in ST data analysis. For the real data-guided pattern, we used the STARmap data as a reference to estimate the required parameters including cell location, cell type proportions and transition matrix and generated spot-level data. For other patterns, we manually set the required parameters to simulate data for the corresponding spatial pattern (see Methods for details).

We first evaluated various spatial clustering methods using simulated data with different patterns. Here, we measured the clustering accuracy based the adjusted Rand index (ARI) (Hubert and Arabie 1985). Overall, BASS achieves better accuracy than other competing methods at both cell- and spot- levels, followed by BayesSpace (Fig. 4B). However, BASS exhibits considerable variability between replicates. Other methods show improved performance for specific patterns, for instance, STAGATE shows higher accuracy in the attractive mode.

Next, we compared different deconvolution analysis methods on the simulated data. To quantify the accuracy of deconvolution, we measured the average correlation between the estimated cell-type compositions and the ground truth across all spots. RCTD consistently outperforms other methods across various datasets and exhibits high reproducibility between replicates, followed by STRIDE (Fig. 4C). In contrast, STdeconvolve displays instability, with significant performance variability across replicates. Importantly, our deconvolution benchmark results using Spider-simulated data are consistent with the conclusions reported in Li *et al.* (Li *et al.* 2022). These findings suggest that Spider can provide a robust benchmark for downstream spatial transcriptomics analyses.

Furthermore, we conducted a systematic comparison of Spider against four other simulation methods (FICT, RCTD, stereoscope, and STRIDE) across both spatial clustering and deconvolution algorithms (Supplemental Fig. S17). Spider consistently demonstrated the highest concordance with real data regarding algorithm ranking patterns. These results confirm that Spider provides a reliable foundation for benchmarking across key spatial analysis tasks, offering a trustworthy reference standard for method evaluation.

### 3.5 Application of Spider in tumor ST data simulation

**Figure 5.**
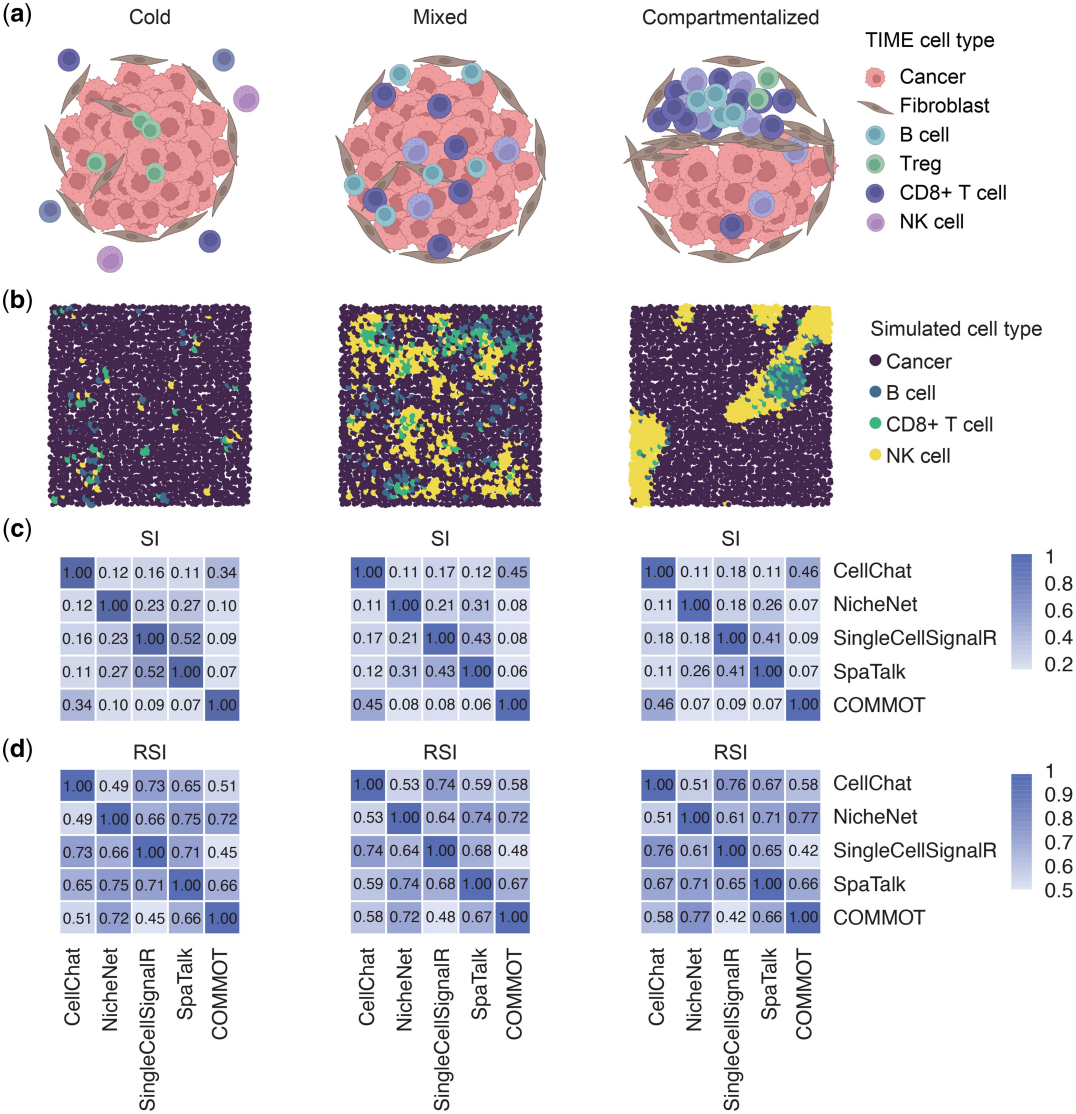
Application of tumor immune microenvironments simulated by Spider. (A) Schematic diagram of three types of TIME in solid tumors (cold, mixed, and compartmentalized). (B) Spider-simulated TIME results by setting proper tumor-immune compositions and cell type transition matrices. (C and D) Heatmaps comparing pairwise SI (C) and RSI (D) metrics between five cell-cell communication tools across three TIMEs simulated using PDAC data. SI, similarity index. RSI, rank-based similarity index.

In this section, we showcased the application of Spider in generating tumor samples with different immune microenvironments (TIME) (Binnewies *et al.* 2018, Fu *et al.* 2021). Previous studies have shown that different tumor cells secrete various cytokines and chemokines, creating a local immune microenvironment by recruiting different types and amounts of immune cells (Ben-Baruch 2003, Atretkhany *et al.* 2016, Nagarsheth *et al.* 2017, Mao *et al.* 2021). The spatial organization of immune phenotypes can be classified into three categories: cold, mixed, and compartmentalized (Keren *et al.* 2018). Immune cold tumors exhibit few infiltrating immune cells, mainly macrophages. In contrast, mixed tumors have numerous immune cells intermixed with tumor cells. Compartmentalized tumors have a high proportion of immune cells, but these cells are spatially segregated from tumor cells (Fig. 5A).

We utilized Spider to simulate these three types of TIME data by setting appropriate cell type proportions and transition matrices (Supplemental Fig. S18). Mixed tumors simulated by Spider reveal a majority of infiltrating immune cells, which are dispersed throughout various tumor regions. Additionally, certain immune cells exhibit aggregation characteristics, closely resembling observed TIME (Fig. 5B). Similar trends are observed in cold and compartmentalized simulated tumors. This result indicates that Spider is an effective tool for simulating diverse tumor-immune spatial landscapes by altering the cellular transition matrix and cell type proportions.

Next, based on the previous parameter settings, we generated cold, mixed, and compartmentalized TIME datasets, each comprising 400 cells and 19 736 genes. Gene expression profiles were sampled from pancreatic ductal adenocarcinoma (PDAC) data (Moncada *et al.* 2020). We employed these TIME datasets to benchmark methods for inferring cell-cell communication (CCC), focusing on interactions among six cell types: cancer cells, T cells, MHCII+ cells, ductal cells, acinar cells, macrophages, and fibroblasts. Two metrics, similarity index (SI) and rank-based similarity index (RSI) (see Methods for details), are used to evaluate five CCC tools: CellChat (Jin *et al.* 2021), NicheNet (Browaeys *et al.* 2020), SingleCellSignalR (Cabello-Aguilar *et al.* 2020), SpaTalk (Shao *et al.* 2022), and COMMOT (Cang *et al.* 2023).

SingleCellSignalR and SpaTalk as well as COMMOT and CellChat show higher SI scores (Fig. 5C), meaning that the two separate pairs of algorithms are more consistent with each other. When focusing on the ranking of overlapping LR pairs within each algorithm, we observed consistently high RSI values between pairwise comparisons of the five CCC algorithms, indicating high consistency in detected LR pairs (Fig. 5D). The consistency between different CCC tools when applied to the TIME data simulated by Spider suggests that our simulation framework can generate biologically meaningful cell-cell communication patterns.

## 4 Discussion and conclusions

In this article, we present Spider, a flexible and comprehensive framework for simulating ST data without requiring real ST data as reference. Spider can generate diverse spatial patterns of cell types and gene expression by leveraging spatial neighborhood patterns among cells via solving a conditional combinatorial optimization problem. Compared with existing simulation methods, a key feature of Spider is its use of cell type proportions and transition probability matrix between adjacent cells to characterize the spatial patterns, resulting in more realistic simulated data and enhanced modeling flexibility. It also provides interactive features for customizing the spatial domain, including zone segmentation, cell coordinate extraction from histology images, and predefined spatial structures.

Benchmarking analyses demonstrated that Spider outperforms other simulation tools in preserving the spatial characteristics of real ST data. Owing to the flexibility of Spider framework, we generated data with diverse spatial patterns to comprehensively evaluate downstream analysis methods, including spatial domain detection and spot deconvolution. Our findings indicate that some methods maintain high performance across various patterns, while others excel only in specific spatial patterns. These results highlight the utility of Spider as a versatile tool for comprehensive algorithm evaluation and as a guide for developing new analytical methods in spatial transcriptomics. Moreover, Spider is capable of simulating ST data for tumor samples with different immune microenvironments, which is difficult for existing simulation tools due to the lack of matched TIME ST datasets. This provides robust support for researchers developing algorithms to study complex tumor ST data, further demonstrating the broad applicability of the Spider framework in spatial biology research.

Spider’s transition matrix-driven approach is inherently agnostic to spatial dimensionality and naturally supports multi-slide extensions. To validate this, we extended Spider to simulate multi-slide ST data for benchmarking cross-condition integration tools like STAligner (Zhou *et al.* 2023). We generated 3D ST simulation data by constructing neighborhood graphs for entire organs, rather than for single tissue sections, and then decomposed the 3D data into sequential 2D slices (Supplemental Fig. S19). Our implementation effectively preserves spatial patterns during dimensional transformations while enabling flexible sectioning along any coordinate axis.

Despite the valuable results, our simulation framework still faces several limitations that should be addressed in future work. First, Spider requires the user to provide a number of parameters, such as cell type proportions and transition matrices, which may not be readily available for all biological systems. This could restrict the utility of Spider in exploratory spatial transcriptomic studies, especially for less-studied biological systems. Second, the current version of Spider focuses on simulating the spatial distribution of cell type patterns, but does not incorporate the temporal dynamics that are often observed in biological systems. This may limit Spider’s applicability in studying dynamic spatial transcriptomic processes, such as tissue development or response to perturbations. Third, Spider’s transition matrix is spatially invariant across tissue regions. As a result, it does not capture boundary dynamics (e.g. meninges-parenchyma junctions) or spatiotemporal pathological evolution (e.g. neurodegeneration or tumor invasion fronts), where transition probabilities vary with spatial coordinates. Finally, Spider does not yet model subcellular mRNA localization or simulate high-density spots within individual cells, as its current version treats cells as discrete units without internal spatial heterogeneity.

Future work should focus on automating parameter estimation from real-world data, further optimizing the simulation algorithms to improve scalability, and expanding the validation and benchmarking using a more diverse set of spatial transcriptomic datasets. Additionally, integrating spatial information from other modalities, such as imaging, spatial proteomics (Lundberg and Borner 2019, Lewis *et al.* 2021, Hickey *et al.* 2022) and spatial metabolomics (Yuan *et al.* 2021), could generate more comprehensive and realistic simulated spatial transcriptomic datasets. By addressing these limitations and exploring new directions, the Spider framework can be further strengthened to become an indispensable tool for the spatial biology research community, enabling more robust evaluation and development of spatial transcriptomic analysis methods.

## Supplementary Material

btaf562_Supplementary_Data

## Data Availability

Spider is a Python package. All codes, simulated ST data in this paper are publicly available at https://github.com/YANG-ERA/Spider. All the datasets analyzed are publicly available from the original publications. The STARmap dataset (mouse visual cortex) is available at https://www.starmapresources.com/data. Smart-seq data (mouse primary visual cortex) is available at https://portal.brain-map.org/atlases-and-data/rnaseq/mouse-v1-and-alm-smart-seq. The human dorsolateral prefrontal cortex datasets on the 10x Visium platform are accessible at https://github.com/LieberInstitute/spatialLIBD. The annotation of mouse olfactory bulb structures is accessible at the Allen Reference Atlas of an adult mouse brain (https://atlas.brain-map.org/). The PDAC data is available at https://www.ncbi.nlm.nih.gov/geo/query/acc.cgi? acc=GSE111672.
