# Supplementary material for: Spider: a flexible and unified framework for simulating spatial transcriptomics data
Source: Bioinformatics. 2025 Nov 14;42(1):btaf562. doi: 10.1093/bioinformatics/btaf562 (PMC12790819; doi:10.1093/bioinformatics/btaf562)
Supplement: btaf562_Supplementary_Data [file btaf562_supplementary_data.pdf]

Supplementary Information

Spider: a flexible and unified framework for simulating spatial transcriptomics data

Jiyuan Yang[1,†], Nana Wei[2,3,4,†], Yang Qu[5], Congcong Hu[6], Weiwei Zhang[6], Lin Liu[7], Hua-Jun Wu[2,3,4,*], Xiaoqi Zheng[8,*]

1 School of Mathematical Sciences, Shanghai Jiao Tong University, Shanghai 200240, China

2 Key laboratory of Carcinogenesis and Translational Research (Ministry of Education/Beijing), Department of Lymphoma, Peking University Cancer Hospital & Institute, Beijing, 100142, China

3 Department of Biomedical Informatics, School of Basic Medical Sciences, Peking University Health Science Center, Beijing 100191, China

4 Center for Precision Medicine Multi-Omics Research, Institute of Advanced Clinical Medicine, Peking University, Beijing 100191, China

5 Department of Mathematics, Shanghai Normal University, Shanghai, China

6 School of Mathematical and Information Sciences, Shaoxing University, Shaoxing, Zhejiang 312000, China

7 Institute of Natural Sciences, MOE-LSC, School of Mathematical Sciences, CMA-Shanghai, SJTU-Yale Joint Center for Biostatistics and Data Science, Shanghai Jiao Tong University, Shanghai, 200240, China

8 Center for Single-Cell Omics, School of Public Health, Shanghai Jiao Tong University School of Medicine, Shanghai, 200025, China

*Corresponding authors: hjwu@pku.edu.cn (HJW); xqzheng@shsmu.edu.cn (XZ)

[†]Co-first authors: Jiyuan Yang and Nana Wei

This PDF file includes:

Figures S1 to S19

Tables S1

Supplementary Figures

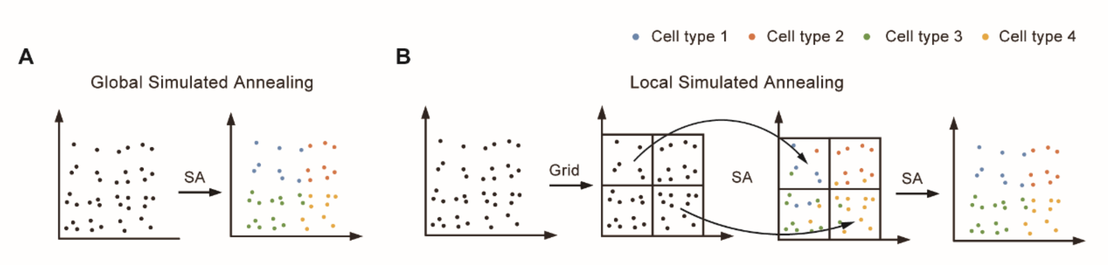

Fig.S1. Detailed illustration of global and local simulated annealing algorithm used in Spider. Global simulated annealing: Spider directly performs the simulated annealing algorithm at the cell-level. Local simulated annealing: cell plate is split into regular grids/layers, with cell type labels randomly assigned to each grid/layer based on the prior cell type proportion. Spider performs the simulated annealing algorithm for each grid/layer in parallel. After convergence, Spider performs the global simulated annealing algorithm to refine the optimization.

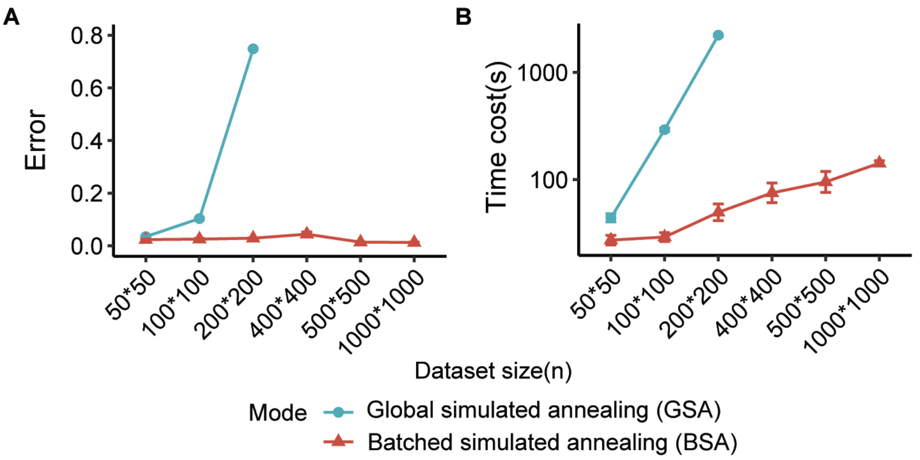

Fig. S2. Performance of different simulation methods with various dataset sizes. **A** Comparison of errors (in terms of transition matrices against reference data) between global simulated annealing (GSA) and batched simulated annealing algorithm (BSA) of Spider. **B** Time costs of two versions (GSA and BSA) of Spider.

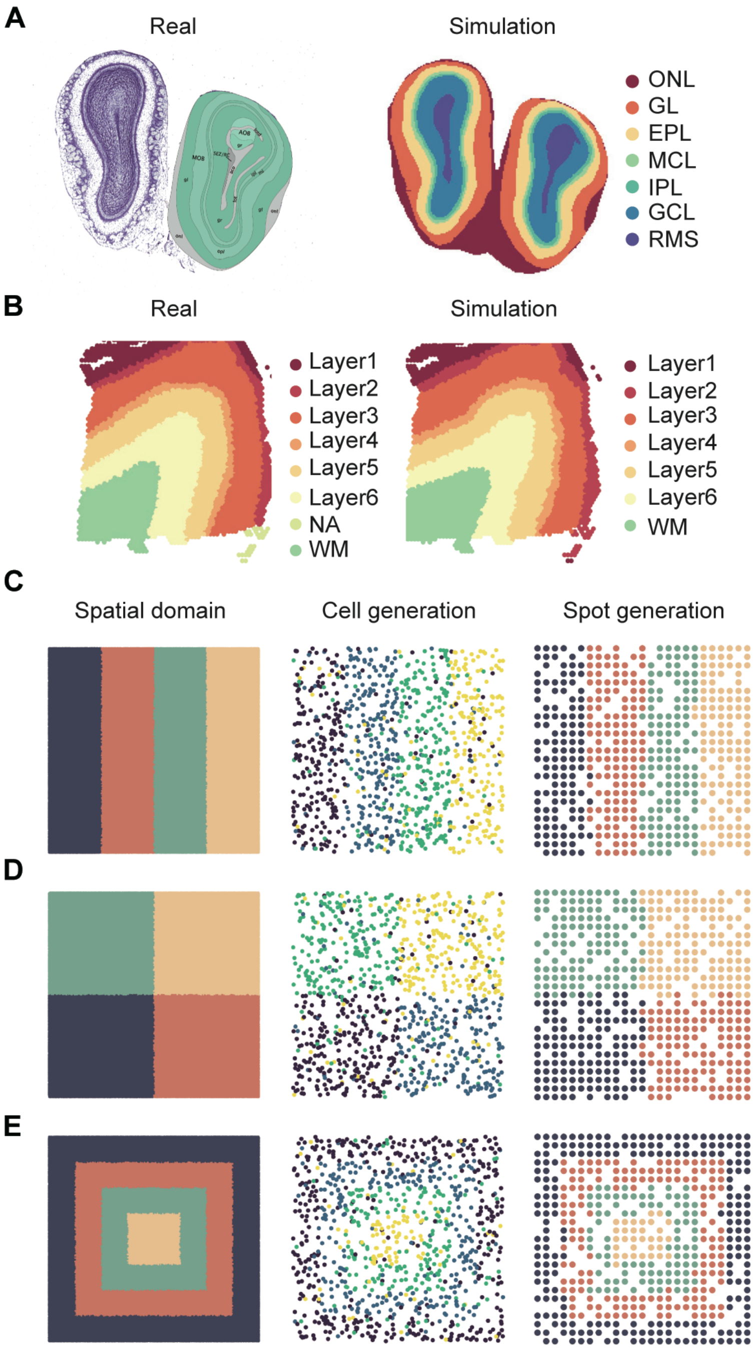

Fig. S3. Example of custom patterns and generated simulation spatial structures by Spider. A Real ST data of mouse olfactory bulb (left) and simulated ST data using interactive module (right). **B** Real spatial structure of DLPFC (left) and the corresponding simulated result using interactive module (right). **C-E.** Three example patterns and simulated results by Spider, i.e., stripe (C), blocked (D) and gyrus (E), including target spatial patterns (left), cell-level simulation results (middle), and spot-level simulation results (right).

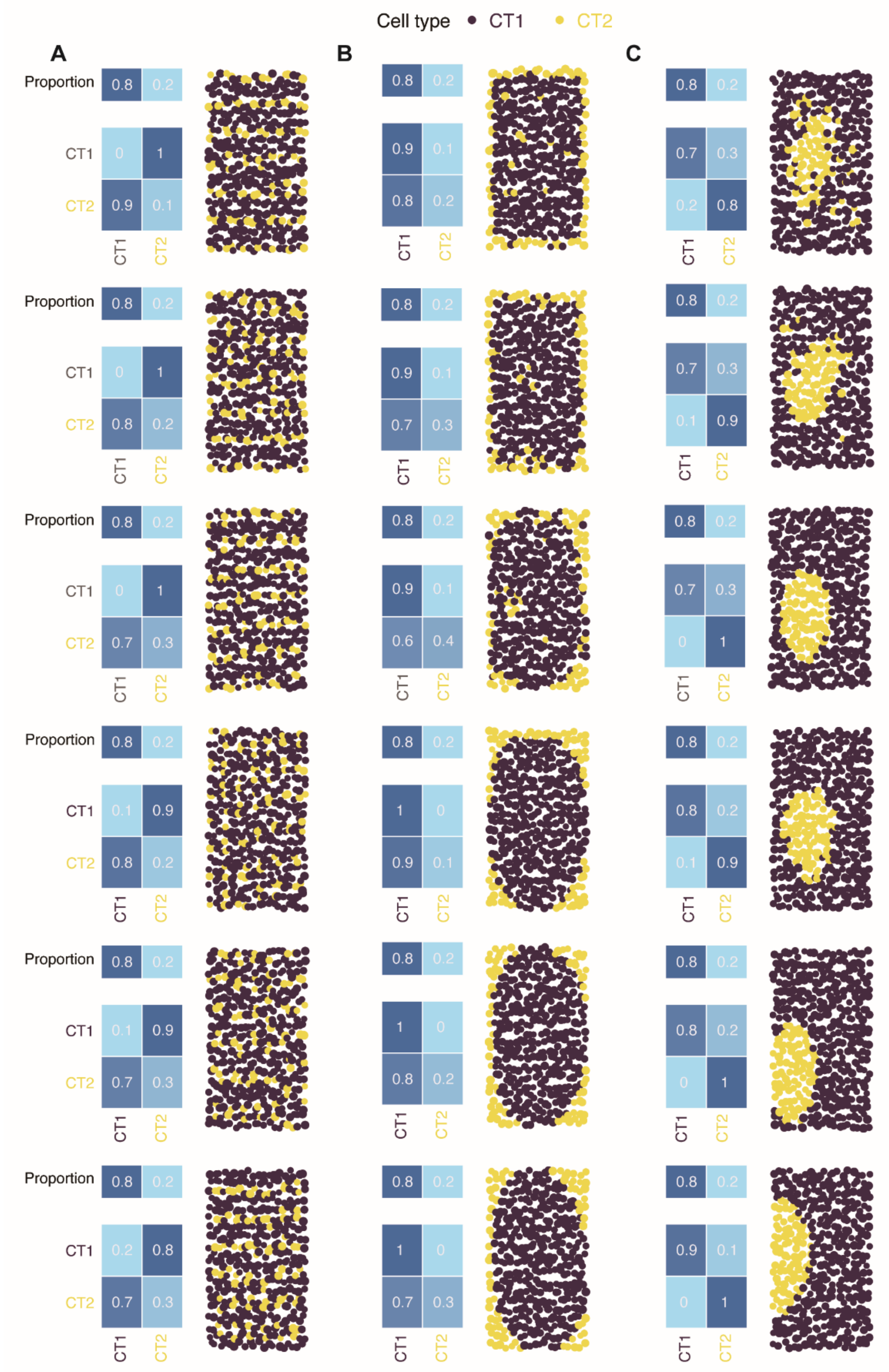

Fig. S4. Examples of simulated data with varying transition probabilities between two cell types. A-C Three distinct distribution patterns corresponding to: mixed where diagonal values remain low across all cell type (A), compartmentalized where diagonal values are high on one cell type and low on another (B) and layered where diagonal values are consistently higher across cell types (C).

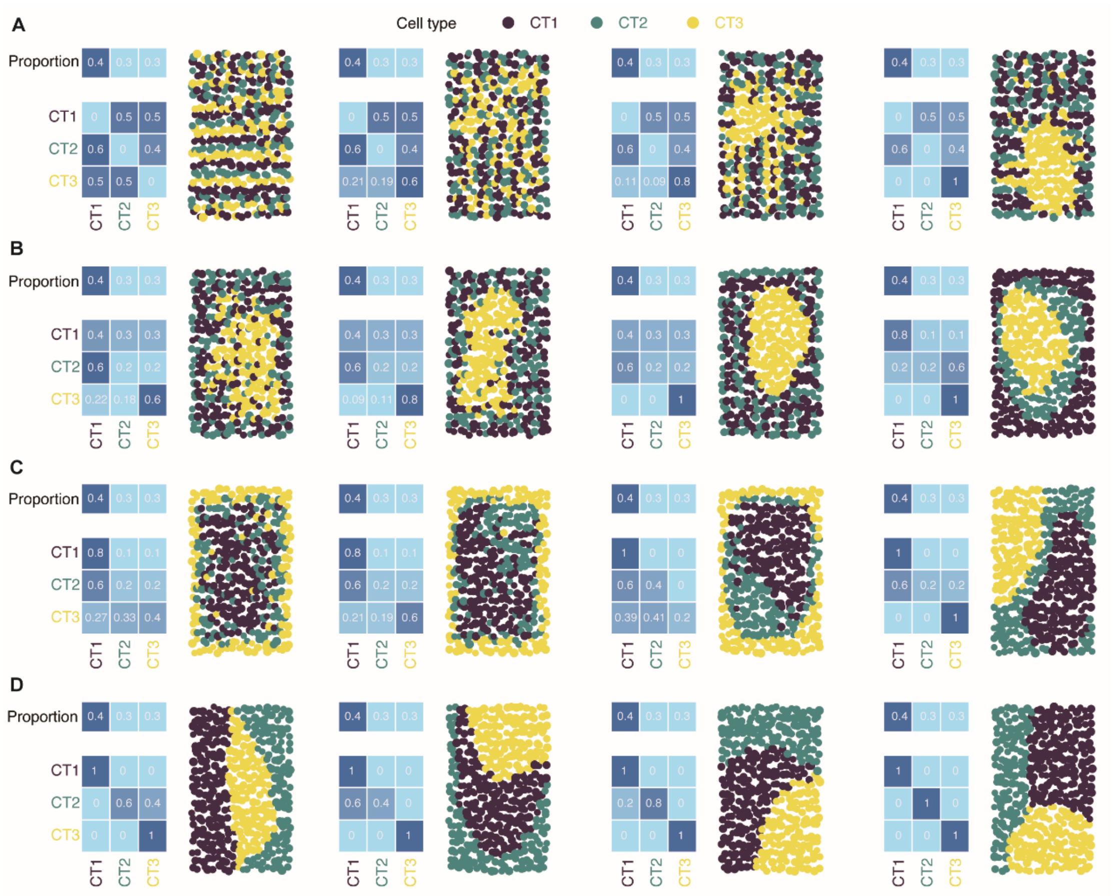

Fig. S5. Examples of simulated data with varying transition matrices among three cell types. A As within transition probability of one cell type increases, the corresponding cells change from a dispersed to aggregated pattern. **B-C** When within transition probability of one cell type is high while the other two are relatively low, it tends to form a surrounding structure. **D** When within transition probabilities of three cells are relatively high, it tends to form a layered structure.

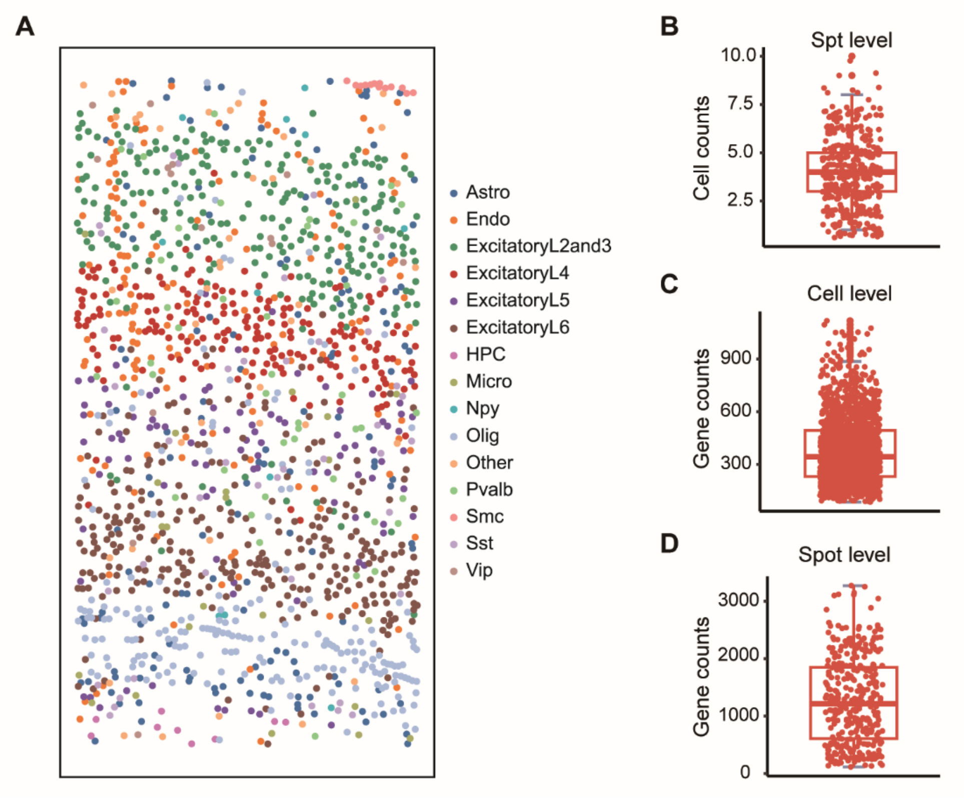

Fig. S6. Summary of cell type annotation and statistics of STARmap data. A Cell type annotation and gridded spot-level ST data. **B** Distribution of cell numbers in each grid (spot-level). **C-D** Distributions of gene counts in cell-level (C) and spot-level (D).

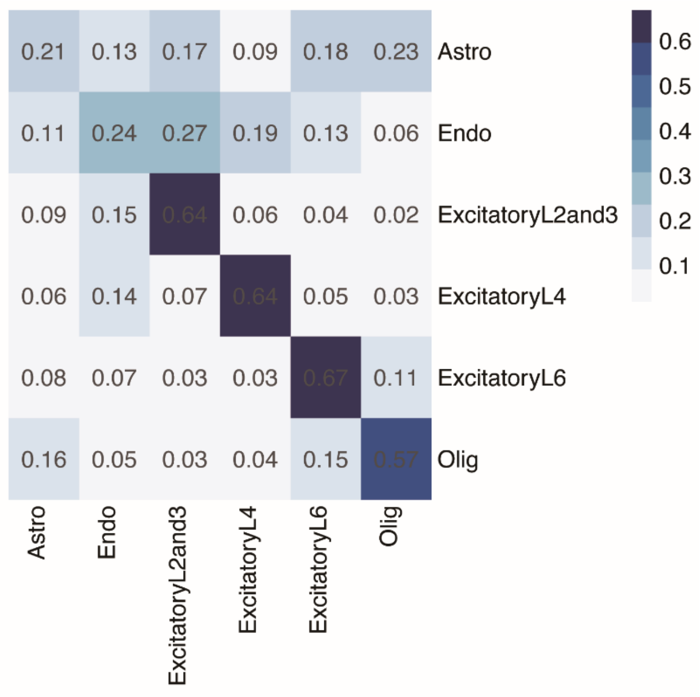

Fig. S7. Cell type transition matrix estimated from the STARmap data.

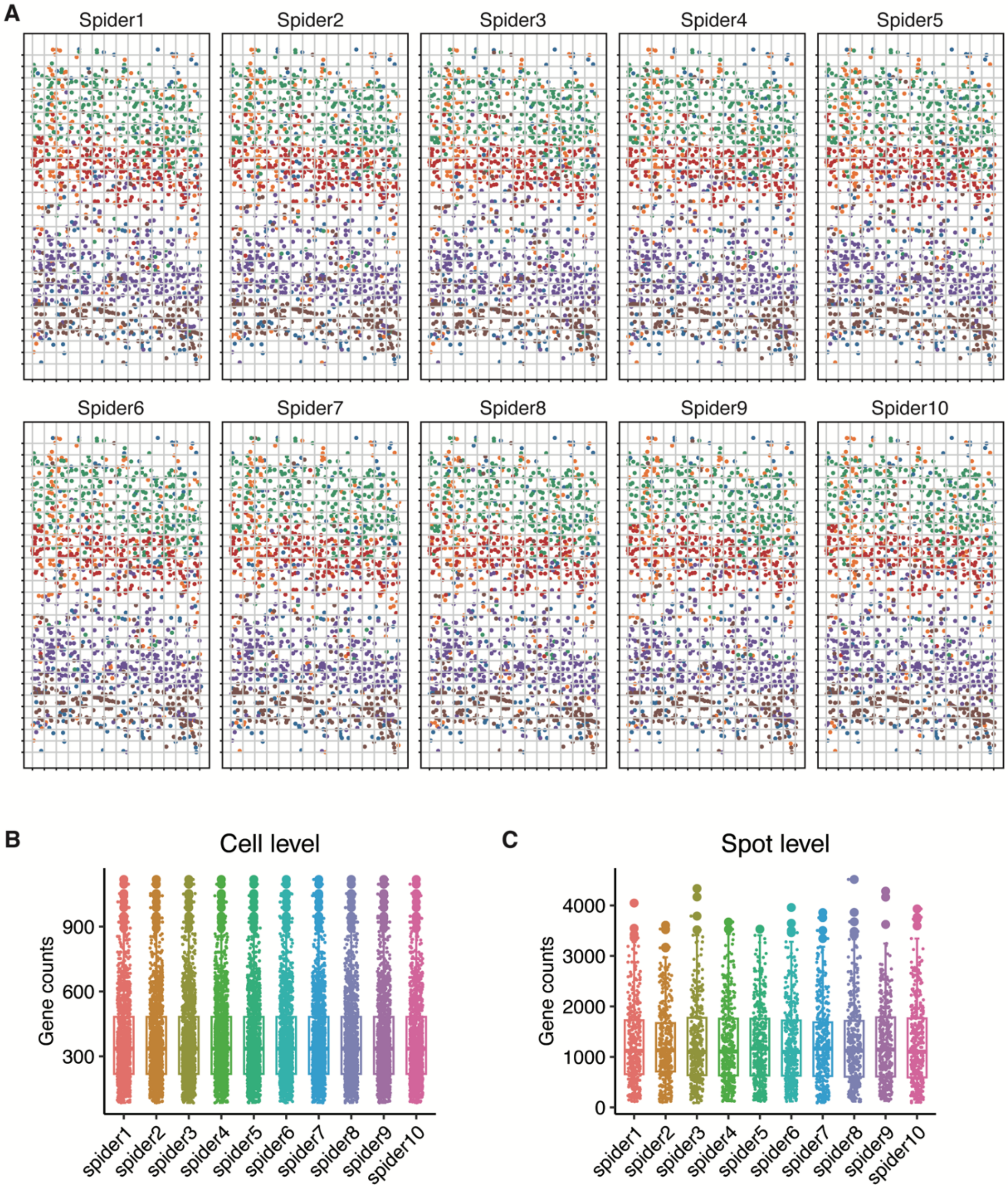

Fig. S8. Simulation results by Spider based on STARmap data. A Distribution of 10 replicate simulations based on cell type proportions and transition matrix estimated from STARmap data. **B-C** Total gene counts of 10 simulated data at cell-level (B) and spot-level (C).

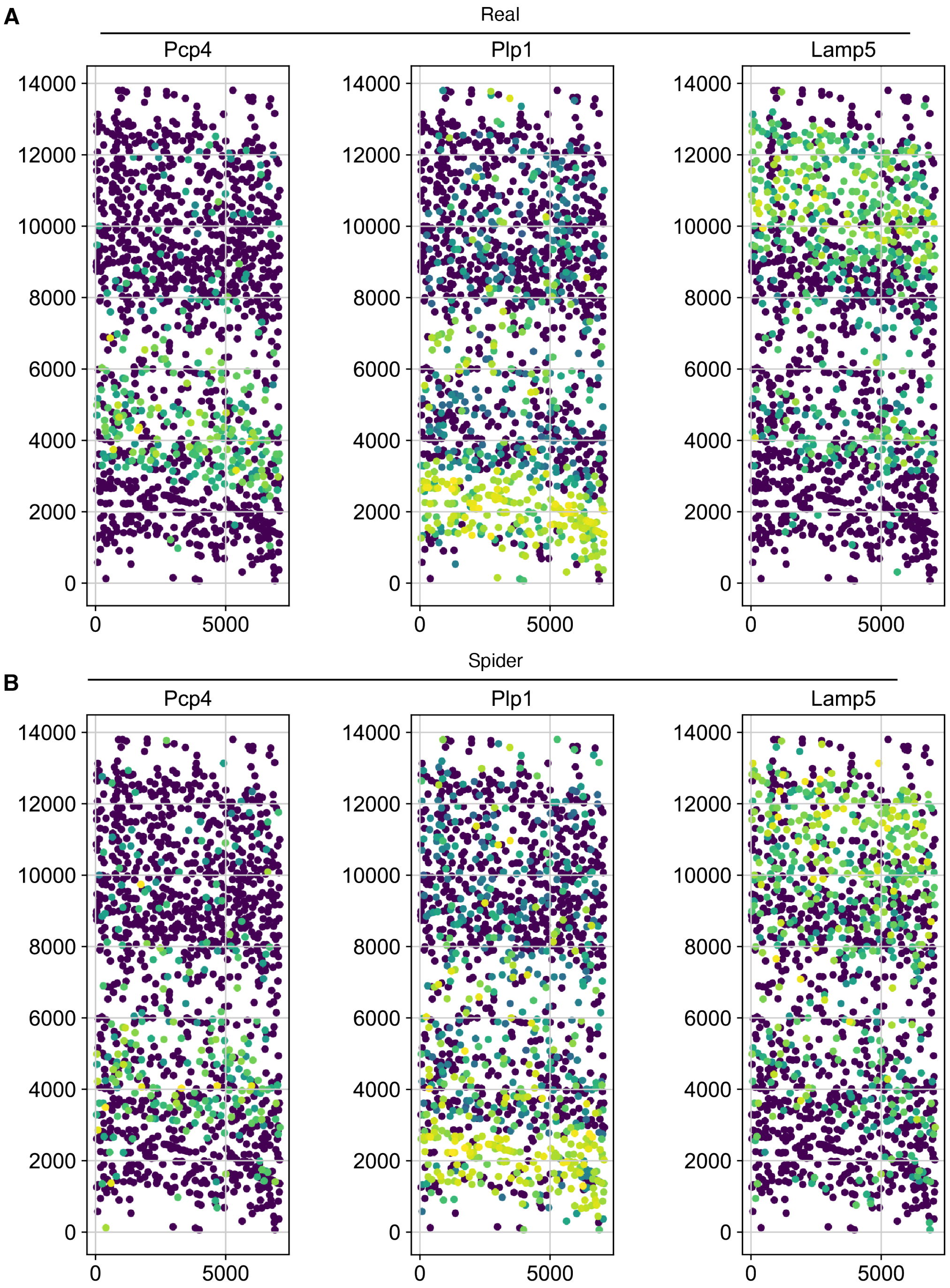

Fig. S9 Spider preserves biologically realistic spatial gene expression patterns. **A-B** Real (A) and Spider-generated (B) spatial transcriptomics data for three marker genes (*Pcp4*, *Plp1*, and *Lamp5*).

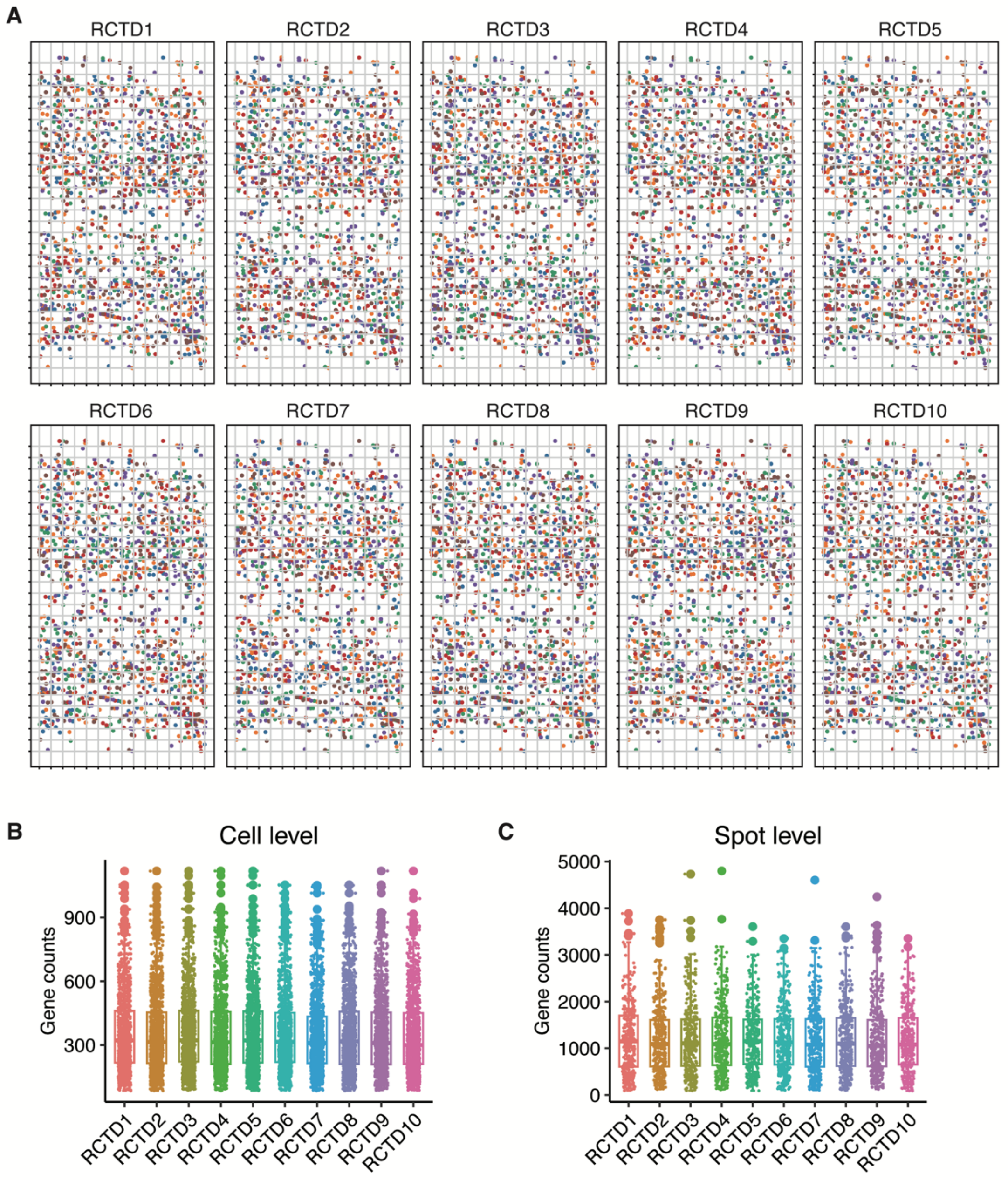

Fig. S10. Simulation results by RCTD based on STARmap data. A Distribution of 10 replicate simulations. **B-C** Total gene counts of 10 simulated data at cell-level (B) and spot-level (C).

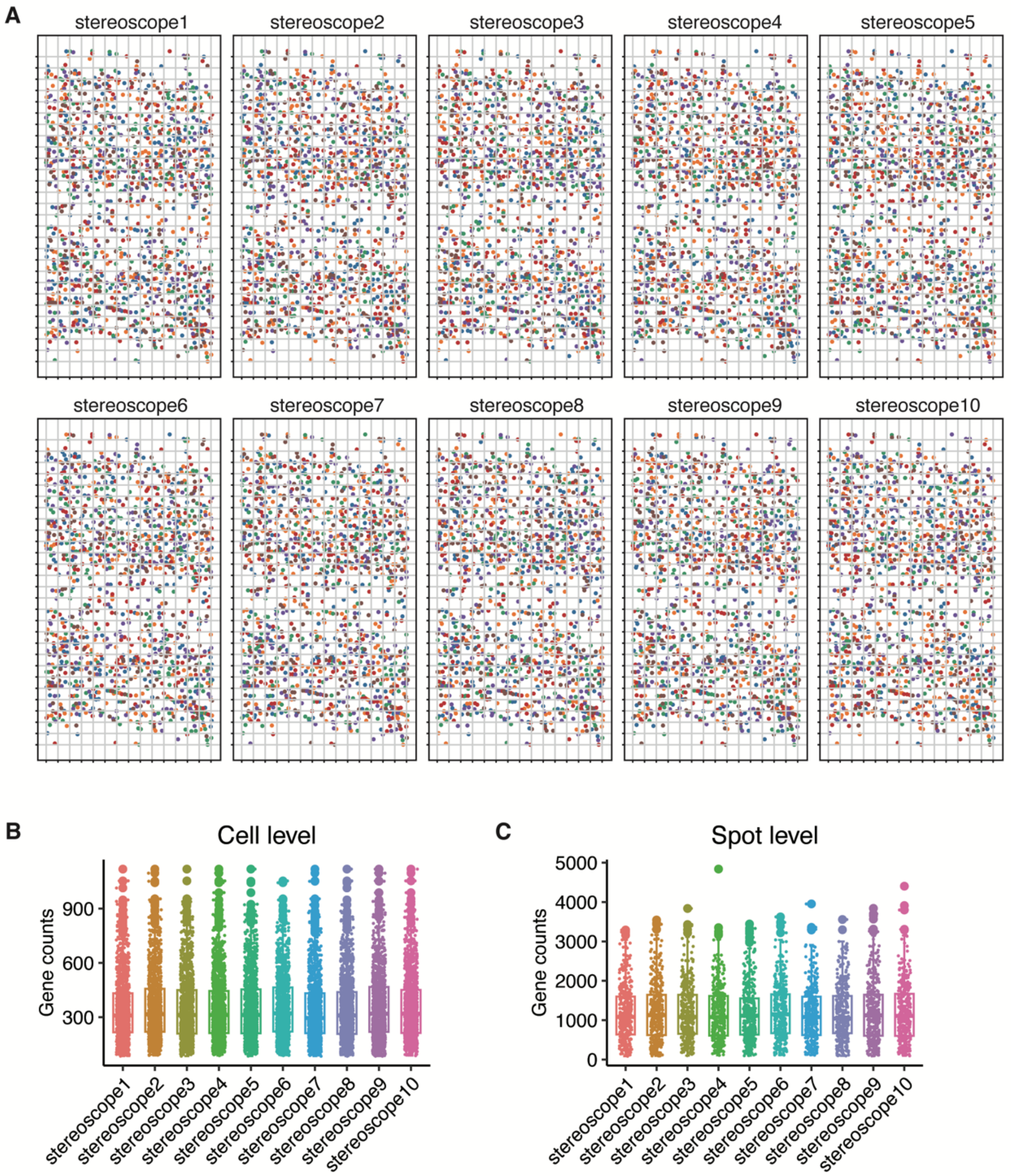

Fig. S11. Simulation results by stereoscope based on STARmap data. A Distribution of 10 replicates. **B-C** Total gene counts of 10 simulated data at cell-level (B) and spot-level (C).

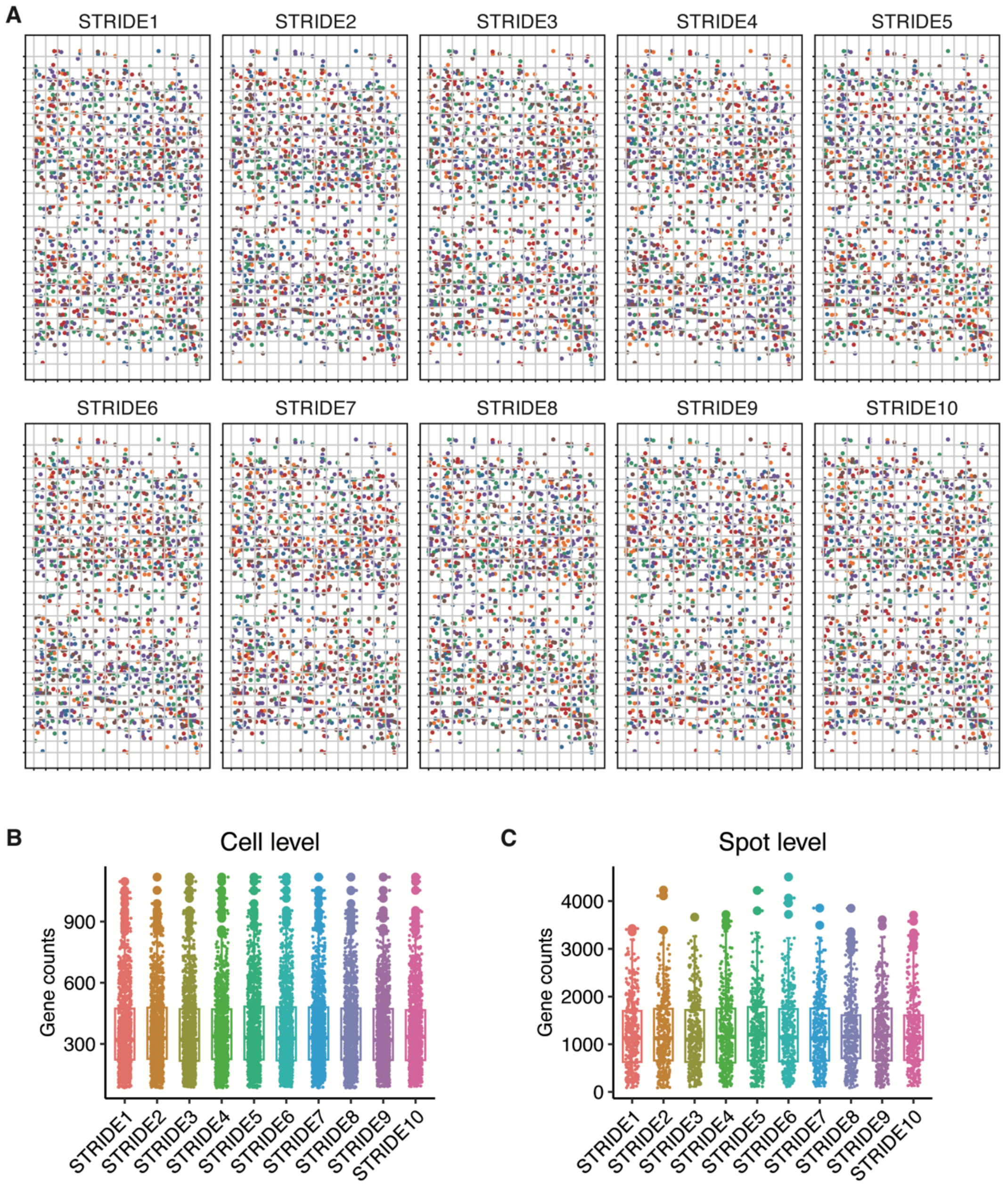

Fig. S12. Simulation results by STRIDE based on STARmap data. A Distribution of 10 replicates. **B-C** Total gene counts of 10 simulated data at cell-level (B) and spot-level (C).

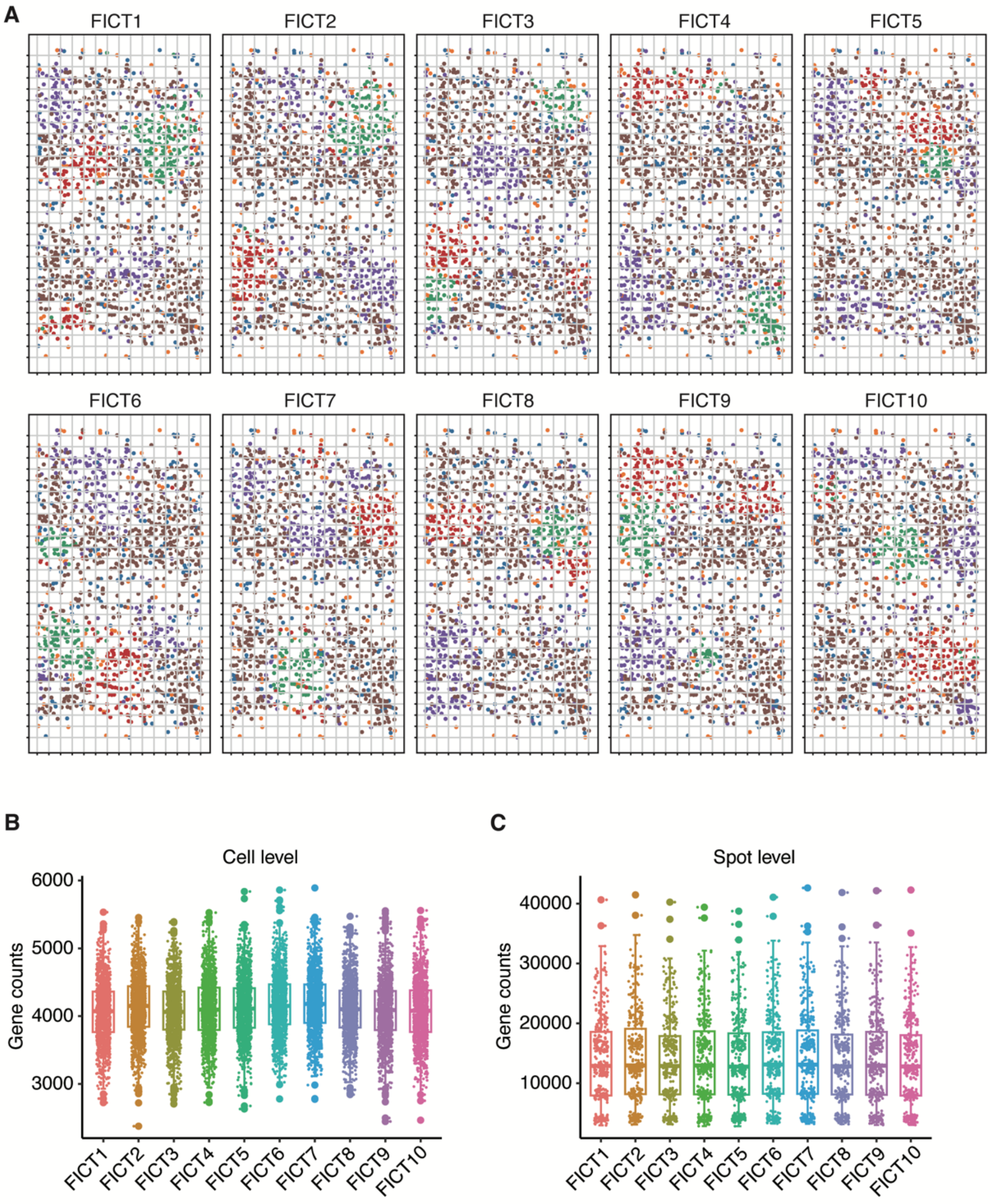

Fig. S13. Simulation results by FICT based on STARmap data. A Distribution of 10 replicates. **B-C** Total gene counts of 10 simulated data at cell-level (B) and spot-level (C).

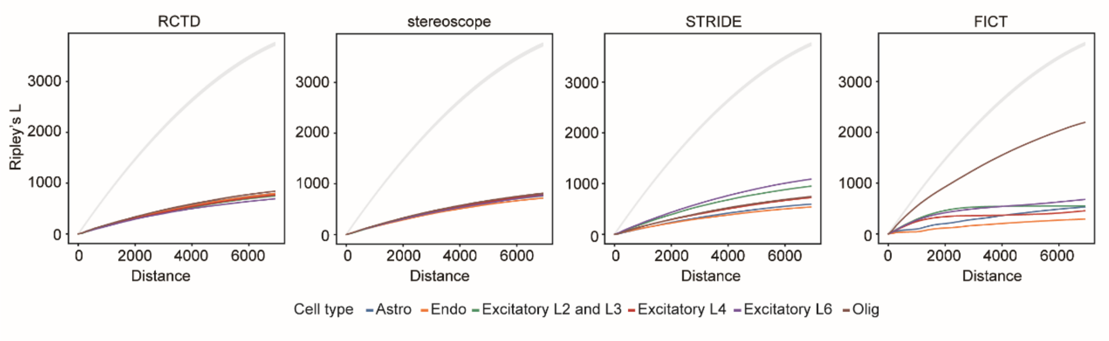

Fig. S14. Ripley's curves of simulated data. Ripley's curves of simulated data generated by various simulation methods including FICT, RCTD, stereoscope, and STRIDE.

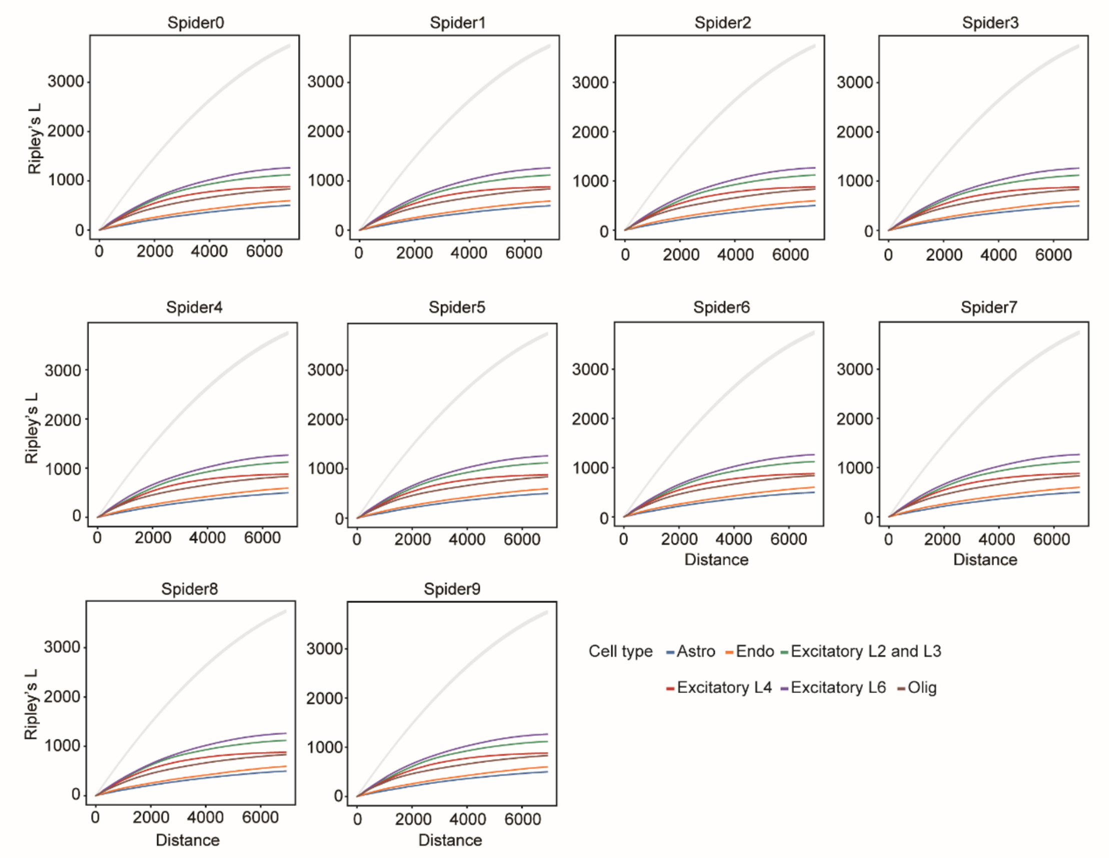

Fig. S15. Ripley's curves of simulated data generated by Spider.

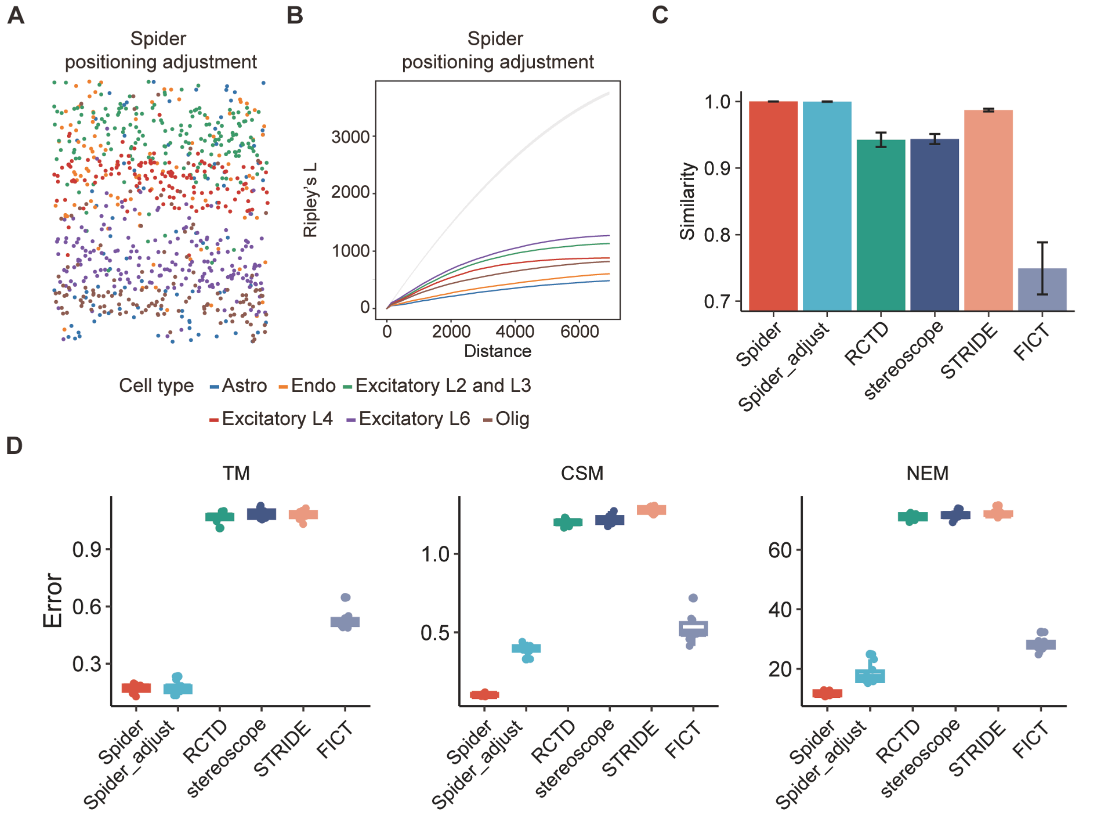

Fig. S16. Sensitivity analysis of Spider based on the STARmap reference data. A Cell type distribution of simulation result generated by Spider with positional adjustment. **B** Ripley's L curves illustrating the global spatial patterns of each cell type in Spider's position-adjusted simulations. The *x*-axis represents the spatial distance at which the Ripley's L function evaluates the spatial pattern of each cell type. The shaded gray line represents the expected L-function of a random pattern under spatial Poisson point process. **C** Cosine similarity of the Ripley's L curve between the reference and simulated data generated by different simulation methods. **D** Differences between real and simulated data in terms of three spatial pattern matrices (i.e., TM: transition matrix, CSM: centrality scores matrix, NEM: neighborhood enrichment matrix). Spider_adjust denotes Spider with positional adjustment.

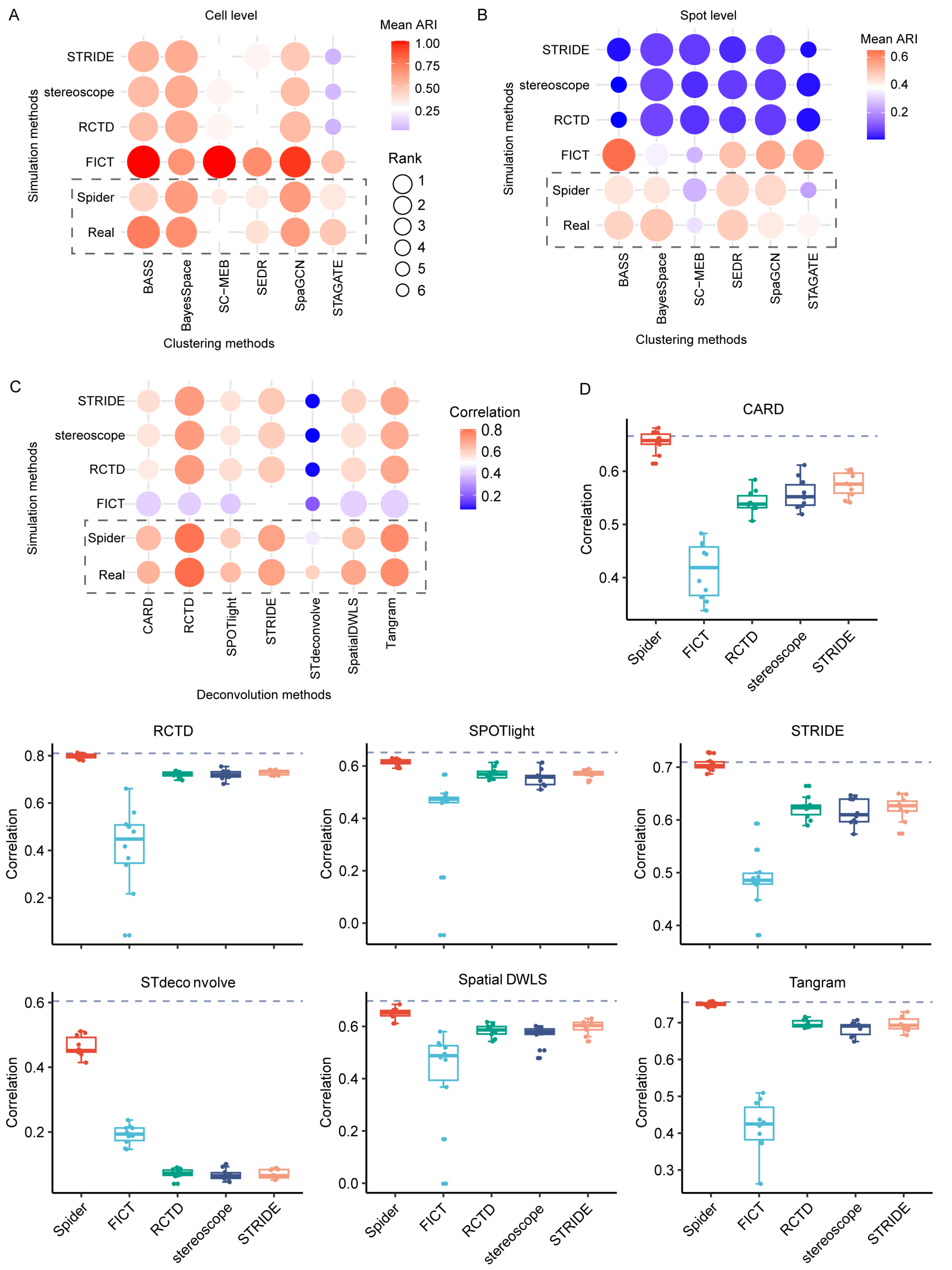

Fig. S17. Benchmarking performance comparison of Spider and other simulation methods across multiple clustering and deconvolution algorithms. A-B ARI matrix for simulation methods (rows) across clustering algorithms (columns) on cell level (A) and spot level (B). Color intensity represents values of ARI. Circle size represents the ranking of clustering methods, with larger circles indicating better performance. Rank information is shown on the left (1-6, with 1 being highest rank). **C** Correlation matrix for simulation methods (rows) across deconvolution algorithms. **D**

Each plot shows correlation distributions from 10 replicates for each simulation method (Spider, FICT, RCTD, stereoscope, and STRIDE). Y-axis represents correlation between predicted and true cell type proportions. Horizontal dashed line indicates performance from real data.

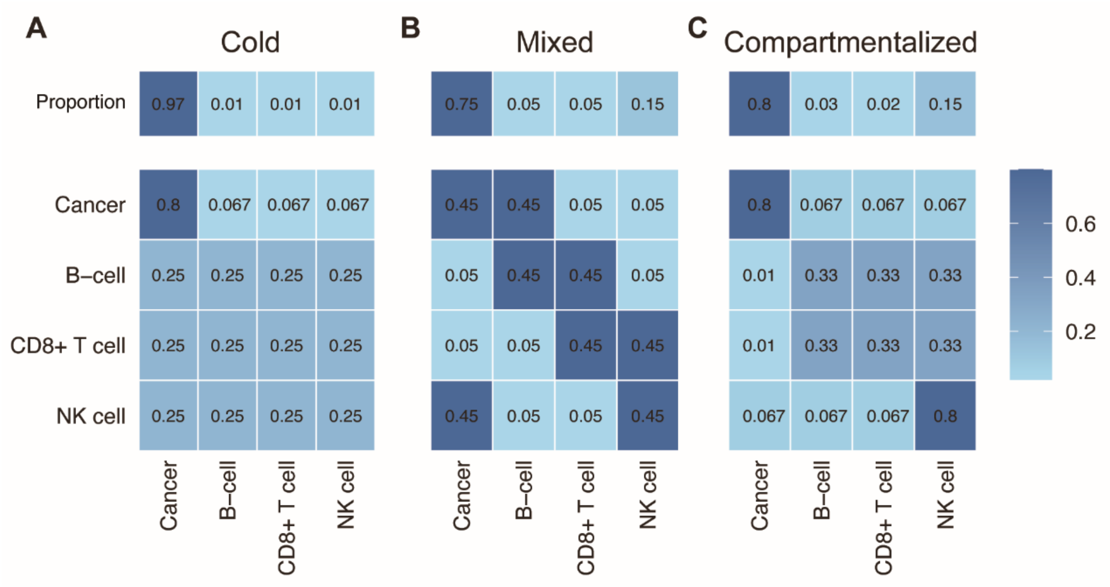

Fig. S18. Cell type proportions and transition matrices for generating different tumor immune microenvironments (TIME) in Fig. 5.

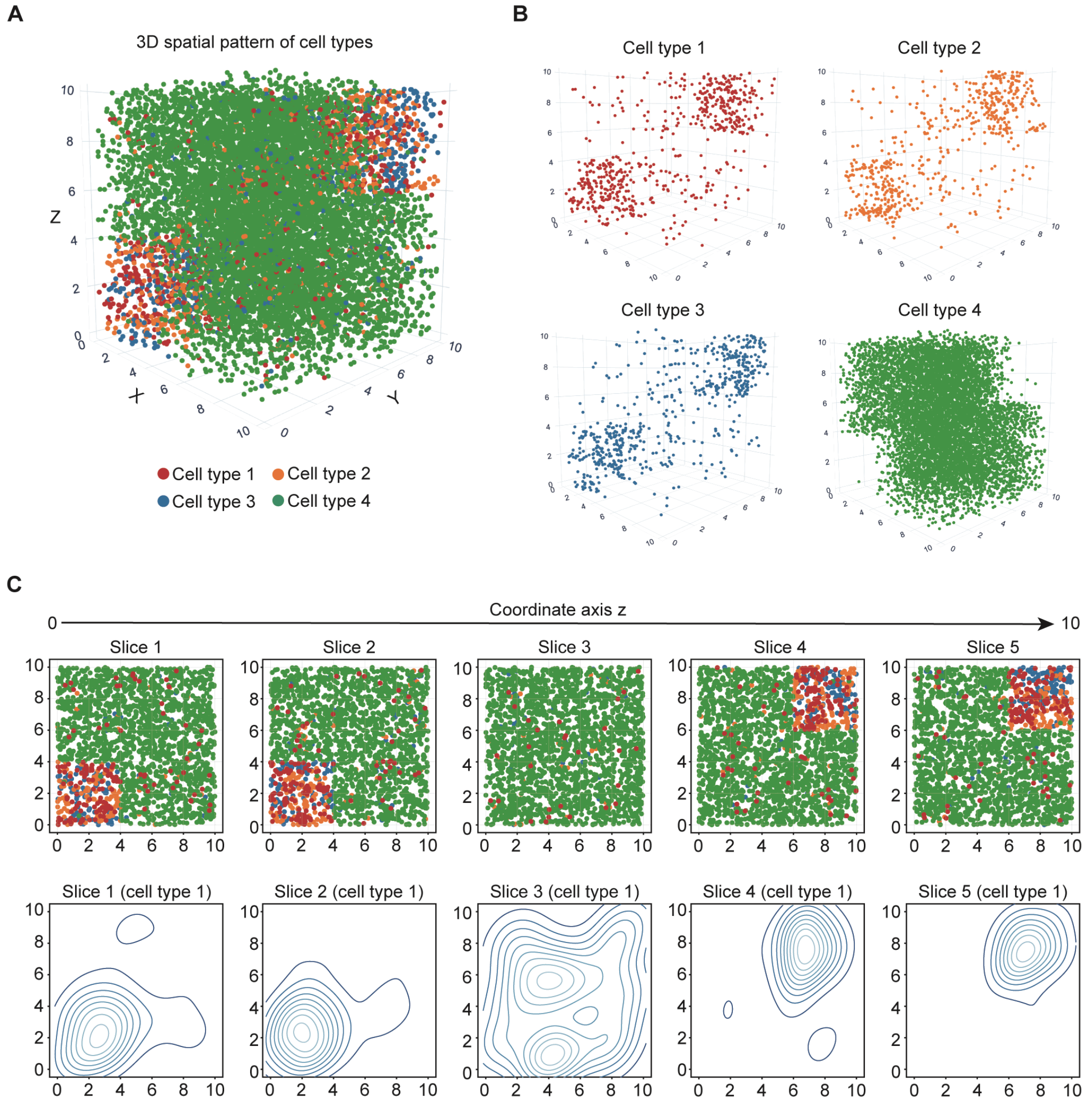

Fig. S19. Spider generates spatial patterns of three-dimensional spatial transcriptomics datasets. A Three-dimensional simulation incorporating four distinct cell types generated by Spider. **B** Spatial distribution of each cell type. **C** A series of two-dimensional slices derived from the three-dimensional simulation results along the *z*-axis. The overview of spatial patterns (top) and the spatial distribution of cell type 1 (bottom) are showed respectively.

Supplementary Tables

Table S1.

Number of cells for each cell type obtained by different simulation methods

	Real	Spider	RCTD	stereoscope	STRIDE	FICT
Astro	141	141	213	213	161	144
Endo	150	150	211	192	143	81
ExcitatoryL2and3	258	258	194	204	251	128
ExcitatoryL4	198	198	204	209	195	118
ExcitatoryL6	287	287	187	200	287	190
Olig	200	200	225	216	197	573
